# Mitochondrial complex I deficiency-associated diseases and models

**DOI:** 10.1007/s00018-026-06169-2

**Published:** 2026-06-10

**Authors:** Lena Jentsch, Natascia Ventura

**Affiliations:** 1 Institute of Clinical Chemistry and Laboratory Diagnostic, Medical Faculty, Heinrich Heine University Düsseldorf, Moorenstraße 5, 40225 Düsseldorf, Germany; 2 IUF – Leibniz Research Institute for Environmental Medicine, Auf’m Hennekamp 50, 40225 Düsseldorf, Germany; 3 Institute of Cell Biology, Heinrich Heine University Düsseldorf, Universitätsstraße 1, 40225 Düsseldorf, Germany; 4 Department for the Promotion of Human Science and Quality of Life, San Raffaele University of Rome, Via di Val Cannuta 247, 00166 Rome, Italy

**Keywords:** Mitochondrial complex I, Mitochondriopathies, Mammalian cell models, Model organisms

## Abstract

Mitochondrial complex I is the first and largest enzyme of the mitochondrial respiratory chain and thus plays a crucial role in cellular energy metabolism. Defects in the mitochondrial respiratory chain, and in particular CI deficiency, are the primary cause of human mitochondrial associated diseases, which most often presents as severe neurometabolic disorders with fatal outcome. Up to this date the diagnosis and treatment of CI deficiency-associated diseases is challenging, only limited symptomatic therapies exist and no cures are available. This review aims at summarizing current knowledge on the genetic basis of CI deficiency-associated diseases and available experimental disease models. Most common human disorders caused by CI deficiency range from Leigh syndrome to MELAS and LHON, all characterized by genetic and symptomatic heterogeneity. So far, *in vivo* studies on non-mammalian organisms and mouse models, as well as *in vitro* studies on patient derived fibroblasts, cybrids and human-induced pluripotent stem cells have mainly facilitated the research of CI deficiency. These model systems provide insights on molecular mechanisms in mitochondrial disease and approaches for potential therapeutic intervention strategies. However, current research is limited by translational relevance of existing disease models, varying degrees of heteroplasmy and tissue specific effects characteristic of mitochondrial diseases, so that basic disease mechanisms still remain poorly understood. To overcome these challenges there is an urgent need for *in vivo* and *in vitro* human relevant models to aid the development of effective therapeutic interventions and potential cures of CI deficiency-associated diseases.

**Supplementary Information:**

The online version contains supplementary material available at 10.1007/s00018-026-06169-2.

## Introduction

Mitochondrial complex I (CI) is an essential component of the respiratory chain and oxidative phosphorylation (OXPHOS) system and plays a critical role in cellular energy production. As an entry point of electrons in the respiratory chain, CI is responsible for a key step in the generation of adenosine triphosphate (ATP). Dysfunction or deficiency in CI function can have severe consequences, leading to a wide range of debilitating uncurable diseases [1].

Mitochondrial CI-associated diseases comprise a heterogeneous group of disorders that primarily affect tissues with high energy demands, such as the brain, heart, and skeletal muscles. These diseases often present with a broad spectrum of signs and symptoms, including neurodegeneration, cardiomyopathy, and metabolic abnormalities [2]. Despite significant advances in our understanding of CI biology, there are no known cures for these devastating disorders and even symptomatic treatments remain limited.

To accelerate the development of therapeutic strategies, it is crucial to enhance our knowledge of the pathogenesis of CI deficiency-associated diseases by developing relevant experimental disease models. Robust disease models that recapitulate the pathophysiology and clinical manifestations are essential tools for unravelling the underlying molecular mechanisms and exploring potential targeted therapeutic interventions. To this end, past efforts mainly focused on cellular *in vitro* systems, but to accelerate therapeutic strategies, in later years more efforts are being revolved towards the establishment of 2D or even 3D *in vitro* systems and *in vivo* models to recapitulate human mitochondria-associated disorders [3, 4].

The purpose of this review is to provide a comprehensive overview of the structure and function of mitochondrial CI within the respiratory chain and OXPHOS system and highlight serious diseases arising from known mutations affecting CI function, which emphasizes the urgent need for the development and utilization of more relevant disease models. Subsequently, we will delve into the existing repertoire of disease models for CI deficiency-associated diseases, critically assessing their strengths and limitations. Overall, the effort to elucidate the underlying molecular mechanisms and pathological consequences of mitochondrial CI-associated disease, along with the development of relevant disease models, are necessary for advancing our understanding of disease pathogenesis and ultimately paving the way for the discovery of effective therapeutic interventions.

## Complex I of the mitochondrial respiratory chain

### Mitochondrial electron transport and OXPHOS

The respiratory chain or electron transport chain (ETC) of the mitochondria is a complex process that arranges a series of reduction and oxidation (redox) reactions, ultimately driving the synthesis of ATP through the OXPHOS system and therefore fulfilling a vital role in cellular energy metabolism. Five major protein complexes along with electron carriers, ubiquinone and cytochrome c, facilitate this process [5].

The first complex, CI or NADH: ubiquinone oxidoreductase, composed by both nuclear and mitochondrial encoded protein subunits, initiates the electron transfer by oxidizing nicotinamide adenine dinucleotide (NADH) to NAD^+^ and transferring two electrons to ubiquinone (Q). Simultaneously, CI pumps four protons across the inner mitochondrial membrane from the matrix to the intermembrane space. This process establishes an electrochemical gradient, with a higher concentration of protons in the intermembrane space [6]. Complex II (CII), or succinate dehydrogenase, contributes electrons to the ETC by oxidizing succinate, a product of the citric acid cycle. The electrons are transferred directly to ubiquinone, bypassing CI. Unlike CI, CII does not pump protons across the inner mitochondrial membrane [5]. The electron transfer then continues with Complex III (CIII), or ubiquinone: cytochrome c oxidoreductase that accepts electrons from ubiquinone and transfers them to cytochrome c. This complex is responsible for the translocation of protons across the inner mitochondrial membrane, playing a role in establishing the electrochemical gradient [5]. Cytochrome c receives electrons from CIII, then delivers them to complex IV (CIV), a cytochrome c oxidase. This enzyme facilitates the final step of electron transport by transferring electrons to molecular oxygen, which serves as the final electron acceptor. The reduction of oxygen leads to the formation of water as a by-product. At the same time, CIV transfers four protons across the inner mitochondrial membrane, contributing to the electrochemical gradient [7]. The resulting gradient, created by the cumulative proton pumping activities of CI, CIII, and CIV, drives the protons back into the mitochondrial matrix through the fifth complex, the ATP synthase or complex V (CV). ATP synthase acts as a molecular turbine, using the energy of the proton gradient to convert adenosine diphosphate (ADP) and inorganic phosphate into ATP [7]. This process is known as chemiosmotic coupling, as the flow of protons drives the mechanical rotation of ATP synthase, enabling the synthesis of ATP [8]. All ETC complexes, except for CII, consist of multiple subunits encoded by both the nuclear and mitochondrial genomes. Their synthesis therefore requires a tightly coordinated crosstalk between these two genetic systems [9].

### Mitochondrial complex I

Electrons derived from NADH enter the mitochondrial ETC through CI, whose core protein subunits are highly conserved due to its central role in cellular metabolism. Consequently, genetic or environmentally-induced disruption of CI is associated with mitochondrial dysfunction. For once, environmental toxicants such as pesticides and micro- or nanoplastic particles have been reported to impair mitochondrial function, often by targeting ETC complexes, including CI [10, 11]. Moreover, mutations in CI subunits or assembly factors account for approximately 30% of all human mitochondrial diseases in childhood [12], underscoring the importance of understanding CI structure and function in the context of CI deficiency-associated disease.

#### Structure of CI

**Fig. 1:**
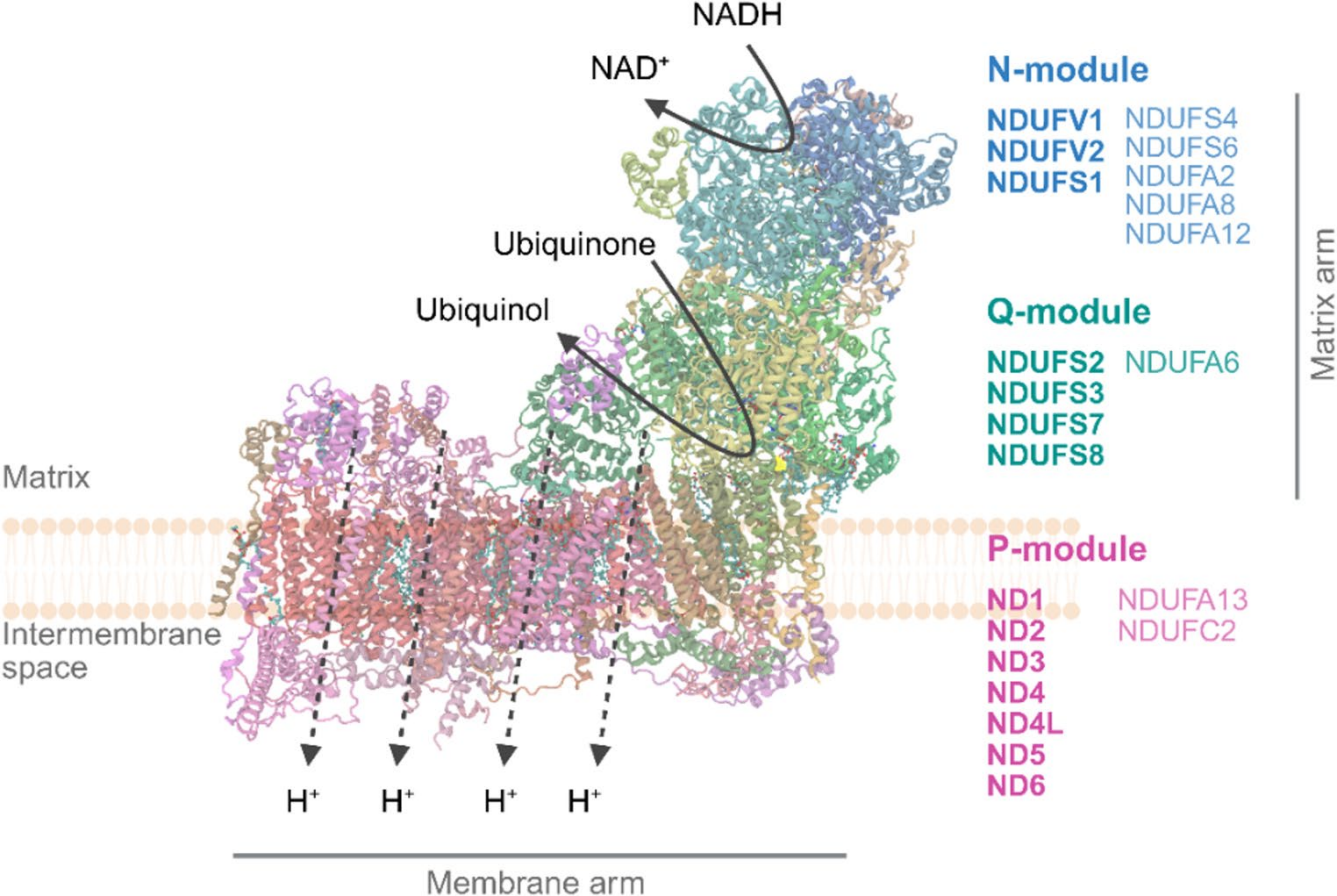
Structure and function of mitochondrial Complex I. L-shaped complex, located in mitochondrial matrix and inner mitochondrial membrane. Consisting of N-module (NADH oxidation), Q-module (ubiquinone reduction) and P-module (proton pumping). CI introduces electrons into the mitochondrial respiratory chain and contributes to the electrochemical gradient, driving the production of ATP at mitochondrial complex V. CI core subunits (bold) and selected accessory subunits, associated with CI deficiency, listed under their respective module. Created in https://BioRender.com with image from the RCSB PDB (RCSB.org) of PDB ID 67KD [33]

The mitochondrial CI (Fig. 1) is the largest membrane protein of the ETC and is a type I NADH dehydrogenase, belonging to the protein family of H^+^ or Na^+^ translocating NADH dehydrogenases [13]. In 1962 the isolation of CI from bovine heart mitochondria and the purification of the enzyme has enabled the analysis of structural aspects and the characterization of individual subunits of CI for the first time [14, 15]. For a long time, the investigation of the structure of CI has been impaired by a lack of high-resolution images. More recent advances however have allowed an increased resolution and gave insight into the structural composition of mammalian CI, a protein with a weight of 1 MDa [16–18].

The L-shaped mammalian CI contains a total of 45 subunits of which 14 are so-called core subunits. These are highly conserved across species [19–21]. CI core subunits are organized into three structural modules. Two hydrophilic modules, called N- and Q-module, form the globular shaped matrix arm located in the mitochondrial matrix, and one hydrophobic module, the P-module, forms the elongated membrane arm in the inner mitochondrial membrane [22]. The seven protein core subunits in the matrix arm are encoded by the nuclear genome, synthesized in the cytoplasm and subsequently imported into the mitochondria [19]. The N-module includes a flavin mononucleotide (FMN) cofactor and an NADH-binding subunit, whilst the Q-module contains a ubiquinone (Q) binding site [9]. The matrix arm of CI contains in total eight iron-sulphur (Fe-S) clusters [23]. Seven of these Fe-S clusters connect the N-module of the matrix arm to the Q binding subunit at the interface with the membrane arm [16]. The N-modules core subunits are NDUFV1, NDUFV2 and NDUFS1 and the Q-modules protein core subunits are called NDUFS2, NDUFS3, NDUFS7 and NDUFS8 [24]. Since there is no uniform nomenclature, these terms refer to protein in mammals whilst terminology in other species e.g. yeasts, bacteria and fungi differs [22].

The membrane arm is formed by the P-module, which is mainly responsible for the translocation of protons by pumping them across the inner membrane of the mitochondria [25], and it also consists of seven core protein subunits, ND1, ND2, ND3, ND4, ND4L, ND5 and ND6 [9]. Differently from the matrix arm, these seven subunits are encoded by the mitochondrial DNA (mtDNA) [26]. The membrane arm of CI spans the inner mitochondrial membrane and consists of several transmembrane helices and Fe-S clusters and contains four putative proton translocation channels [17]. The protein subunit ND1 forms the reduction site for Q, whilst the other core subunits are believed to be involved in proton translocation [27]. The 14 core subunits of mammalian CI are joined by numerous accessory subunits which stabilize the enzyme and play a role in the protection against reactive oxygen species (ROS) [9, 28]. Functional aspects of CI’s core subunits and the whole enzyme in context of the respiratory chain will be described in more detail in the following chapter, but many features of the core and especially accessory subunits still remain unknown.

#### Function of CI

CI has an important role in the mitochondrial respiratory chain hence it has major influence on the energy household of a cell. As the first protein complex CI introduces two electrons into the respiratory chain. The N-module forming part of the matrix arm shows dehydrogenase activity and is responsible for the electron input by accepting electrons from NADH while the Q-module shows hydrogenase activity and is responsible for the electron output by delivering electrons to Q [19]. Thus CI firstly binds and subsequently oxidizes NADH to NAD^+^ at the NADH binding site, by donating two electrons to the initial acceptor FMN, which is placed in the N-modules core subunit NDUFV1 in the peripheral matrix arm [29]. The NDUFV2 subunit, closely located to the FMN, is believed to control the access of the substrate NADH to prevent overreduction of CI and thus might control ROS production [30]. The two electrons resulting from the oxidation of NADH are then passed through an arrangement of eight Fe-S clusters and are transferred to Q in its oxidized form. Q then takes up two electrons and forms ubiquinol. When transferring the electrons from one redox centre to the next, four protons from the matrix are pumped through CI into the mitochondrial inter membrane space [9]. Putative proton translocation pathways for this transfer are built by the core subunits ND1, ND3, ND4L and ND6 in the membrane arm of CI close to the Q site. ND2, ND4 and ND5 show similarities in structure to cation/H^+^ antiporters and are believed to hold proton channels [5, 27]. The exact mechanism of coupling the redox reactions occurring in CI with its proton pumping activity remains unknown. However, it is believed that conformational changes of the P-module, caused by the reduction of Q, induce the proton pumping [31]. Moreover, the function of CI cannot be considered in isolation, as it is now well established that the respiratory chain protein complexes interact to form supercomplexes that contribute to overall respiratory function [32].

## Genetics and clinical manifestations of CI deficiency

Disruption of mitochondrial CI primarily affects tissues with high energy demand. Clinical manifestations of CI deficiency vary widely, from severe lactic acidosis and early infant death to adult-onset muscular weakness. CI deficiency can be associated with seven main clinical categories: Leigh syndrome (LS) and Leigh-like syndrome (LLS), progressive leukoencephalopathy or mitochondrial encephalomyopathy with lactic acidosis and stroke-like episodes (MELAS), neonatal cardiomyopathy, severe infantile lactic acidosis, Leber’s hereditary optic neuropathy (LHON), and various undefined encephalomyopathies [19, 34–37]. LS is an early-onset, progressive neurodegenerative disorder caused by mutations in either nuclear or mitochondrial genes [38, 39]. Hallmarks include bilateral, often symmetrical brainstem lesions with vacuolated neuropil, demyelination, gliosis, and capillary proliferation [34]. Despite similar neuropathological features, LS shows high genetic and clinical heterogeneity. Typical symptoms include developmental delay, hypotonia, spasticity, ataxia, seizures, nystagmus, cardiac involvement, and elevated lactate levels [38, 39]. Diagnosis of LS is commonly based on stringent criteria as defined by Rahman et al.: (1) progressive neurological disease with motor and cognitive delay; (2) brainstem and/or basal ganglia involvement; (3) elevated lactate in blood or cerebrospinal fluid; and (4) characteristic radiological or neuropathological findings. However, LS has also been reported without lactic acidosis [40]. Patients with suggestive features but not meeting full LS criteria are classified as LLS [34, 41].

Another CI deficiency-associated clinical phenotype is MELAS syndrome, characterized by transient cortical brain lesions on neuroimaging [42]. MELAS syndrome is mitochondrially inherited and often displays heteroplasmy or variability in the mtDNA of different tissues mostly affecting skeletal and heart muscle, eye, inner ear and brain [43]. The clinical features are stroke-like episodes before age 40, encephalopathy with seizures or dementia, and lactic acidosis or ragged-red fibres [44].

A further neuropathy caused by mutations of mtDNA and associated with CI deficiency is LHON [37]. It is among the most common mitochondrially inherited optic neuropathies, leading to subacute, painless central vision loss typically in early adulthood [45, 46].

Mutations have been reported in numerous CI subunits, including all 14 core subunits. Representative pathogenic variants and key clinical features of all core subunits and further accessory subunits as well as experimental model systems developed so far to study the associated diseases are listed in Table 1. Details on specific pathogenic variants and associated clinical features are listed in Supplementary Table 1.

### Mutations affecting N-module

Pathogenic variants in genes encoding both core and accessory subunits for CI’s N-module are associated with a wide spectrum of clinical phenotypes, highlighting the marked heterogeneity of CI deficiency.

Multiple pathogenic variants in core subunit *NDUFV1* have been identified predominantly in cases of severe LS and LLS. Bénit et al. reported heterozygous *NDUFV1* mutations including point mutations and deletions in patients with isolated CI deficiency, presenting with brainstem and basal ganglia atrophy and lactic acidosis [47]. Additionally, lethal infantile mitochondrial disease with comparable phenotypes have been linked to homozygous and heterozygous *NDUFV1* point mutations, characterized by early-onset LS, with lactic acidosis, brainstem lesions, and seizures [47–50]. Interestingly, also less severe LS-phenotypes linked to variants of *NDUFV1* have been described [51]. Beyond LS and LLS, homozygous and heterozygous point mutations of *NDUFV1* have also been linked to more heterogenous symptomatology including leukoencephalopathy, hypotonia, ataxia, spasticity or paralysis [36, 50, 52, 53].

Similar clinical features are also commonly associated with homozygous and heterozygous point mutations of the core subunit *NDUFV2*, including leukoencephalopathy and severe early-onset encephalopathy [54–56]. The disease onset is typically early and progresses rapidly with fatal outcome. *NDUFV2* variants have also been reported in cases of severe cardiomyopathy [57].

Among N-module subunits, the core subunit *NDUFS1* is one of the most frequently affected genes with heterogenous point mutations and deletions generally associated with severe clinical phenotypes of mitochondrial disease. Homozygous and heterozygous point mutations, as well as deletions in *NDUFS1* have been associated with (leuko-)encephalopathy, optic atrophy and LLS [47, 58], but also with severe LS and leukoencephalopathy accompanied by lactic acidosis and hypotonia [36, 59–64].

In addition to core subunits, several accessory N-module subunits play essential roles in CI assembly and function. Accordingly, pathogenic variants in the accessory subunit *NDUFS4* are strongly associated with LS, with homozygous and heterozygous point mutations and deletions reported in patients with severe LS and LLS, but are also linked to cardiomyopathy [48, 57, 65, 66]. Similarly, pathogenic variants of accessory subunit *NDUFS6* are commonly associated with severe, early-onset clinical phenotypes, most notably LS, fatal lactic acidosis and encephalopathy [67–70]. Rarer pathogenic variants affecting subunits *NDUFA2* and *NDUFA12*, including homozygous and heterozygous point mutations, deletions or insertions, are associated with early-onset LS and leukoencephalopathy [71–74], in isolated cases they can even be linked to isolated optic atrophy or scoliosis [72]. Variants of *NDUFS8* are generally linked to a broad range of severe neurological symptoms, ranging from developmental delay, early onset hypotonia and epilepsy to cerebellar atrophy and microencephalopathy [36, 75].

Although overall pathogenic variants affecting the N-module are frequently associated with severe, early-onset and fatal neurological diseases, which are characterised by a set of different but generally similar clinical features, the severity and prognosis vary considerably [69, 76, 77]. For example, pathogenic *NDUFV1* variants causing lactic acidosis and encephalopathy may also cause milder phenotypes with facial paralysis, muscle weakness, and poor balance. Remarkably, even full recovery of motor milestones within three years after symptom onset has been reported [76, 77]. Prognosis is further complicated not only by the marked heterogeneity of clinical features but also by significant diagnostic challenges. Biochemical markers such as CI enzymatic activity vary across tissues: some patients exhibit reduced CI activity in muscle and liver but not in fibroblasts [47], whereas others show normal muscle activity but impaired CI activity in fibroblasts [49]. Overall, reduced CI activity is most frequently detected in muscle cells or skin fibroblasts where CI assembly defects are also commonly observed [55, 66, 74, 75, 78]. However, these biochemical features do not consistently correlate with clinical severity [72, 73].

### Mutations affecting Q-module

In contrast to pathogenic variants of the N-module, variants of Q-module subunits are not predominantly associated with severe neuropathologies but commonly display more diverse clinical features. Homozygous and heterozygous point mutations and deletions affecting *NDUFS2* have been reported in cases of LS, presenting with optic atrophy and hypotonia as well as isolated cardiomyopathy and in rare cases liver pathology [79–81]. Here, too, the heterogeneity of clinical features in CI deficiency needs to be emphasized. Indeed, even the same variant of *NDUFS2* was reported to cause varied severity of symptoms and correspondingly very different prognosis in patients with LS and LLS [80] and the broad spectrum of mitochondrial disorders associated with *NDUFS2* do not appear to be specific to any mutation profile [81]. Similarly, pathogenic variants of the core subunit *NDUFS3* caused by heterozygous point mutations are also connected to diverse presentations of LS [82–84] and pathogenic variants of core subunit *NDUFS7* are linked to the clinical presentation of cardiomyopathy without the involvement of the central nervous system but also in patients with LS [36, 85]. Accessory subunits in the Q-module of CI that can be linked to CI deficiency, are for example *NDUFS8* and *NDUFA6*. *NDUFS8* has been the first nuclear encoded subunit to be established as a cause for LS [86]. Since then, additional homozygous and heterozygous point mutations within *NDUFS8* have been identified as causes of CI deficiency. These mutations are associated with encephalopathy and mitochondrial disease with diffuse clinical features, including stroke-like episodes, hypotonia and brain atrophy [48, 87]. Also, pathogenic variants of *NDUFA6* due to homozygous and heterozygous point mutations are associated with diffuse clinical features including lactic acidosis, hypotonia or optic atrophy [88]. Pathogenic variants affecting Q-module overall display high phenotypic variability regarding key clinical features and also the severity of diseases. Johnstone et al. for example have underlined this with reportedly atypical late-onset and mild cases of LS associated with pathogenic variants of *NDUFS3* in contrast to otherwise severe cases of LS [83, 84]. As observed for variants of N-module subunits, diagnosis of CI deficiency caused by pathogenic variants in Q-module subunits is complicated by variable and often inconclusive biochemical parameters, such as lactate levels and CI enzyme activity, which depend strongly on sample type [80, 87, 88]. For instance, in some cases CI activity in muscle cells appears normal despite clear defects in CI assembly and the presence of clinical features of mitochondrial disease [88], further underscoring the challenges of correlating genotype, biochemical findings and clinical phenotype in CI deficiency.

### Mutations affecting P-module

Unlike N- and Q-module, the core P-module subunits are mitochondrially encoded. Generally, pathogenic variants of mtDNA encoded subunits often present with heterogenous symptoms of neurological disease similar to CI deficiency linked to nuclear mutations. However, mtDNA variants are often associated with LHON and with MELAS syndrome. Pathogenic variants affecting subunit *ND1* have been linked to LHON and MELAS syndrome in many cases and are rarely also occurring in late-onset cases of dystonia [89–93]. Pathogenic variants of *ND2* are associated with LHON as well [94]. However, pathogenic variants of *ND1*,* ND3*, *ND4* and *ND5* can also be linked to cases of LS, distinguished from MELAS syndrome by characteristic symmetric brain lesions in the brain stem [34, 36, 95]. Further on, pathogenic variants of *ND3* are also associated with very heterogenous clinical features including LLS, lactic acidosis and diffuse encephalopathy with hypotonia and seizures [96, 97], whereas pathogenic variants of *ND4*, *ND4L* and *ND5* are otherwise primarily associated with late onset MELAS [98] or LHON [94, 99, 100]. In subunit *ND6* pathogenic variants present with very diverse clinical features, comprising of LHON, MELAS and LS [36, 101–104] but also very mild cases, displaying mild and late-onset LHON [104], exercise intolerance and mild myopathy [105].

Pathogenic variants of nuclear encoded accessory P-module subunits are rare and typically associated with neuropathologies. Rare variants of *NDUFC2* caused by heterozygous point mutations have been found in cases of LS [106], whereas more frequent pathogenic variants in *NDUFA13* caused by heterozygous point mutations and deletions lead to moderate to severe clinical features of neuroregression, hypotonia and epilepsy [107]. Overall mutations affecting P-module and especially mtDNA encoded subunits commonly present as multisystemic disorders, with very heterogenous clinical phenotypes. For instance, isolated cardiomyopathy and diabetes can be linked to CI deficiency due to pathogenic variants of *ND2* [94, 108, 109]. As for example Barone et al. have reported, in rare cases pathogenic *ND5* variants are even linked to adult-onset nephropathy [99]. The severe clinical phenotypes caused by pathogenic variants of P-module subunits are often reflected in diagnostic, biochemical analysis, generally displaying decreased CI enzyme activity and defects in CI protein assembly [93, 105, 107].

Although correlations between genotype and phenotype are generally weak in mitochondrial disease, there is one notable exception in LHON. The majority of LHON cases are associated with three recurrent mtDNA mutations: 3460G > A in *ND1*, 11778G > A in *ND4* and 14484T > C in *ND6* [110]. These highly prevalent variants are typically present in homoplasmic state [111] and have been implicated in synergistic interactions with additional mitochondrial mutations [110, 112]. Such synergistic effects are hypothesized to destabilize CI structure, thereby exacerbating functional impairment and contributing to more severe phenotypes [112]. Moreover, similar interactions have been suggested for individually non-pathogenic mtDNA variants, such as certain *ND4L* variants, which, combined with other mtDNA CI variants, may contribute to the penetrance of LHON [113].

**Table 1:** Mitochondrial CI genes with reported, representative pathogenic variants, corresponding key clinical features and established model systems, ordered according to the subunits location in the CI modules

Gene	Representative pathogenic variants	Key clinical features	Available model systems
N-module			
*NDUFV1*	Heterozygous and homozygous point mutations; Deletions [47–50, 53, 76, 77]	LS; LLS; Lactic acidosis; Leukoencephalopathy; Hypotonia; Ataxia; Paralysis	*C. elegans* [114] *Drosophila* [115] Fibroblasts [116] hiPSCs [117]
*NDUFV2*	Heterozygous and homozygous point mutations; Splice-site intronic deletion [54–56]	Lactic acidosis; Cardiomyopathy; Encephalopathy; Loss of motor function; Developmental delay	Not reported
*NDUFS1*	Heterozygous and homozygous point mutations; Deletions [36, 47, 58–62, 64]	LS; LLS; Lactic acidosis; Leukoencephalopathy; Hypotonia	*C. elegans* [118, 119] *Drosophila* [120] Fibroblasts [116] hiPSCs [121]
*NDUFS4*	Heterozygous and homozygous point mutations; Splice-site intronic point mutation; Deletions [57, 60, 65, 66, 78]	LS; LLS; Cardiomyopathy; Encephalopathy	*C. elegans* [118, 122] *Drosophila* [115] Mice[123, 124] Fibroblasts [125, 126] hiPSCs based models [127–131] Human continuous cell line [131]
*NDUFS6*	Heterozygous and homozygous point mutations [67–70]	LS; Lactic acidosis; Encephalopathy; Respiratory failure	Mice [132]
*NDUFA2*	Heterozygous point mutations; Deletion [71, 74]	LS; Cardiomyopathy; Leukoencephalopathy	Not reported
*NDUFA8*	Heterozygous and homozygous point mutations [36, 75]	Hypotonia; Epilepsy; Microcephaly; Developmental delay	Not reported
*NDUFA12*	Heterozygous point mutations; Deletion; Insertion [72, 73]	LS; LLS; Lactic acidosis; Scoliosis; Optic atrophy	Fibroblasts [73]
Q-module			
*NDUFS2*	Heterozygous and homozygous point mutations; Small deletions [36, 79–81]	LS; Lactic Acidosis; Cardiomyopathy; Hypotonia; Epilepsy; Optic atrophy; Hepatopathy	*C. elegans* [133, 134] *Danio rerio* [135] Mice [134] Fibroblasts [136] Human continuous cell line [131]
*NDUFS3*	Heterozygous point mutations [82–84]	LS	*C. elegans* [119] Mice [137]
*NDUFS7*	Heterozygous point mutations; Deletion; Insertion [36, 85]	LS; Cardiomyopathy	*Drosophila* [115] Fibroblasts [116] Human continuous cell line [138]
*NDUFS8*	Heterozygous and homozygous point mutations [48, 64, 86, 87]	LS; Lactic Acidosis; Encephalopathy; Respiratory failure; Loss of motor function; Stroke-like episodes	*Drosophila* [139] Fibroblasts[116]
*NDUFA6*	Heterozygous and homozygous point mutations; Deletions [88]	Lactic acidosis; Hypotonia; Motor regression; Optic atrophy	*C. elegans* [119]
P-module			
*ND1*	Point mutations; Insertions [90–92, 94, 95, 105, 110, 112]	LS; MELAS; LHON; Hypotonia; Loss of motor function; Hearing loss; Neuronal regression	Fibroblasts [140] Cybrids [110]
*ND2*	Point mutations [94, 108, 109]	LHON; Cardiomyopathy; Diabetes mellitus	*Drosophila* [141] Cybrids [142]
*ND3*	Point mutations [36, 67, 96, 97]	LS; LLS; MELAS; Encephalopathy 75	Fibroblasts [140] hiPSCs [143, 144]
*ND4*	Point mutations [36, 92, 98, 110, 112, 145]	LS; MELAS; LHON; Hypotonia; Hearing loss; Neuronal regression	Fibroblasts [36] hiPSCs [146] Cybrids [110]
*ND4L*	Point mutations [113, 145]	LHON	Cybrids [113]
*ND5*	Point mutations [36, 94, 99, 147]	LS; LHON; Nephropathy	Fibroblasts [148] hiPSCs based models [122, 144, 149–152] Cybrids [147]
*ND6*	Point mutations; Deletion [36, 92, 102–105]	LS; MELAS; LHON; Myopathy	Mice [153] Cybrids [113, 154, 155]
*NDUFA13*	Heterozygous point mutations [107]	Hypotonia; Epilepsy; Neuronal Regression	Not reported
*NDUFC2*	Heterozygous point mutations; Deletions [106]	LS	Not reported

### Factors contributing to phenotypic heterogeneity in CI deficiency

As underlined so far, the relationship between genotype and phenotype in CI deficiency remains poorly defined and only few variants show robust genotype-phenotype associations [110, 112]. In general, pathogenic variants in nuclear encoded CI subunits more frequently cause neurological disorders, whereas variants of mtDNA encoded subunits result in a broader spectrum of organ-specific or multisystemic diseases [47]. Mutations of nuclear encoded subunits, particularly affecting the N- and Q-modules of the CI matrix arm are predominantly associated with neurological phenotypes [3, 47, 50, 75]. LS and LLS are most common, alongside isolated neurological features, such as lactic acidosis, ataxia, or hypotonia. Cardiomyopathy is less often a primary manifestation [54, 71, 79], and organ-specific phenotypes including optic atrophy, hepatopathy, or scoliosis are rare [72, 81]. In contrast, pathogenic variants in mtDNA encoded CI subunits, affecting the P-module of CI membrane arm, are linked to more heterogenous clinical presentations [3]. These most commonly include LHON and MELAS [90–92], but also cardiomyopathy [108], diabetes mellitus [109] and nephropathy [99]. Although MELAS is classically maternally inherited, its phenotype can overlap with LS leading to the designation MELAS/LS overlap [97]. Furthermore, pathogenic mtDNA variants can directly cause LS and other encephalopathies as well [36, 92]. So overall, no consistent association can be established between the affected CI subunit, its structural location, or mode of inheritance and the resulting clinical phenotype. This complexity is further exacerbated by marked variability in severity of clinical features among individuals carrying mutations in the same CI subunit, complicating prognostic predictions.

One widely established factor contributing to the phenotypic heterogeneity is the mitochondrial heteroplasmy [156]. Each cell contains multiple thousands of copies of mtDNA, a condition referred to as polyplasmy. Due to limited repair capacity and proximity to ROS, mtDNA exhibits a mutation rate 100–1000-fold higher than nuclear DNA. Newly arising mutations therefore coexist with wildtype mtDNA, resulting in heteroplasmy. Random segregation of mtDNA during cell division leads to a considerable variability in mutant load across cells and tissues [111]. Many mitochondrial disorders, including CI deficiency, exhibit a threshold effect, whereby cellular dysfunction occurs only once mutant mtDNA exceeds a critical level, which varies by tissue and individual. Several studies support a correlation between heteroplasmy levels and disease severity, particularly in LHON [94, 99]. For example, mutations affecting Q-module subunit *ND6* cause LHON at low mutant load level but lead to early-onset dystonia at high levels [157]. Thresholds of critical mutation load range from 60% and > 80%, resulting in phenotypes such as LHON, LS or cardiomyopathy [111, 152, 158]. However, heteroplasmy is not the only contributor to phenotypic heterogeneity in CI deficiency, as the mutant load does not consistently correlate with clinical severity [159]. Experimental models in *Caenorhabditis Elegans (C. elegans)* or *Drosophila melanogaster* demonstrate that mitochondrial dysfunction can be tolerated or even beneficial below a critical metabolic threshold, which is influenced by factors beyond heteroplasmy [156, 160]. Additional support for the existence of such threshold comes from the proposed synergistic interaction of pathogenic mtDNA variants affecting P-module subunits, which are associated with LHON [110, 112]. Compensatory mechanisms preserve cellular function below a certain threshold, and likely contribute to the phenotypic variability in CI deficiency. In mammalian systems, mitochondrial dysfunction triggers adaptive responses, including increased glycolysis, mitochondrial biogenesis and antioxidant defenses [161]. Altered levels of Ca²⁺, NADH, AMP and ROS activate stress signaling pathways, induce mitochondrial biogenesis, notably via PGC-1–dependent pathways, and activate oxidative stress responses mediated by NF-κB, NRF-2 and p53, as well as the upregulation of antioxidant enzymes. In parallel, other mitochondrial quality control mechanisms such as DNA repair, protein turnover, mitophagy or apoptosis limit cellular and organismal damage [156, 162]. Together, these mechanisms contribute to the mitochondrial threshold effect and may help explain tissue-specific vulnerability of CI deficiency and thus the variability of clinical manifestations. Differences in OXPHOS-derived energy demand and metabolic thresholds further contribute to tissue-specific susceptibility to CI deficiency rendering the brain, optic nerve and skeletal muscle particularly vulnerable [3, 158]. Moreover, the phenotypic impact of mtDNA mutations can be strongly influenced by nuclear encoded factors, including CI subunits or assembly proteins, regulators of mitochondrial biogenesis and quality control and potentially retrograde signalling [163]. Combined with broader genetic background effects [164], these nuclear-mitochondrial interactions modulate functional thresholds and contribute to inter-individual variability in disease penetrance and severity, with important consideration for clinical interpretation and experimental modelling of CI deficiency-associated diseases.

## Complex I deficiency disease models

### *In vivo* model organisms

**Fig. 2:**
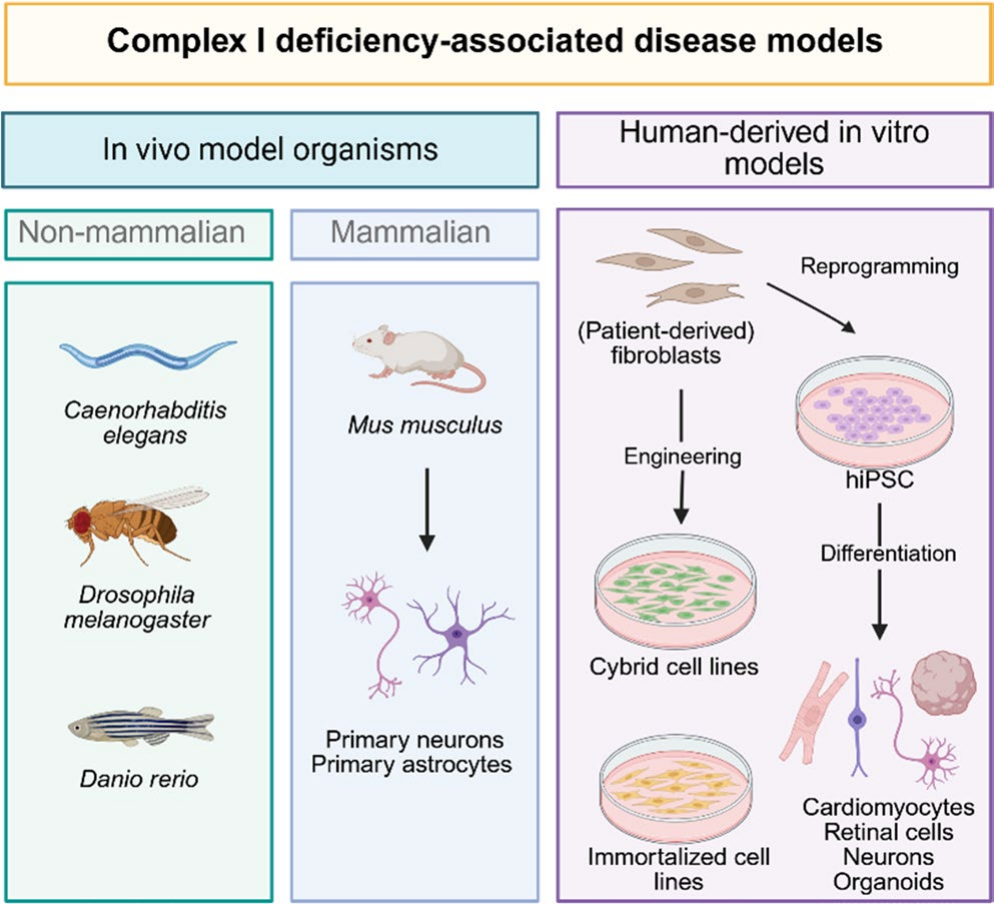
Disease models for CI deficiency-associated diseases. Non-mammalian *in vivo* model organisms include the nematode *Caenorhabditis elegans*, the fruit fly *Drosophila melanogaster* and the zebrafish *Danio rerio*. Mammalian *in vivo* models are available in mouse (mus mu*sculus*), including mouse derived primary cells such as neurons and astrocytes. Human-derived *in vitro* models include patient-derived fibroblasts, human induced pluripotent stem cells (hiPSCs), cybrid cell lines and immortalized continuous cell lines. hiPSCs can be reprogrammed from fibroblasts or engineered and are available in undifferentiated state and differentiated into 2D and 3D models like cardiomyocytes, retinal ganglion cells, neurons and brain organoids. Created in https://BioRender.com

**Fig. 3:**
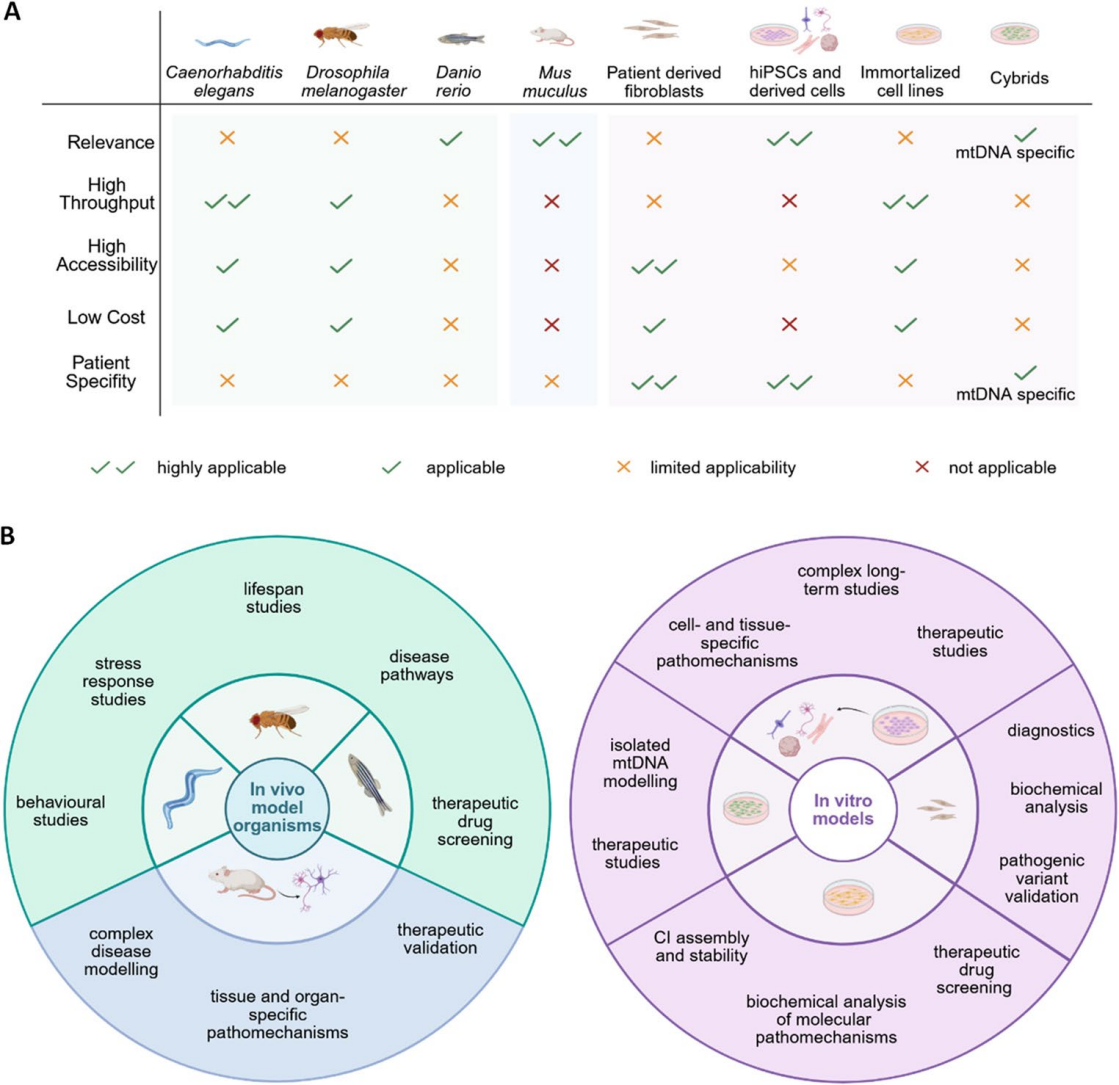
Comparison of main available CI deficiency disease models. **A** Table of most important experimental advantages and trade-offs in CI deficiency disease model selection and; **B** most suitable application fields based on strengths and limitations of *in vivo* model organisms (green non-mammalian, blue mammalian) and *in vitro* models (purple). Created in https://BioRender.com

To facilitate our understanding of CI deficiency-associated diseases pathomechanistic events and advance the development of targeted therapeutic approaches, the generation and use of relevant *in vivo* and *in vitro* model systems recapitulating phenotypic and behavioural features of the mammalian disorders is of vital importance. The following section reviews latest advances of experimental model systems primarily exploited for studies of molecular mechanisms underlying CI deficiency-associated diseases, diagnostic and clinical implications as well as development of new possible therapeutic options (Table 1; Figs. 2 and 3).

#### *C*. *elegans*

*C. elegans* is a soil nematode and a widely used invertebrate model organism for mitochondrial disease research. It has a short life cycle of 3–4 days at 20 °C, fully sequenced and highly conserved nuclear and mitochondrial genome, and structured and functionally conserved mitochondria as well as the majority of mitochondrial quality control and stress response pathways [11] compared to humans. Consequently, different *C. elegans* models of CI deficiency have been generated over the past decades, using RNA interference (RNAi) or genetic engineering to target multiple CI subunits [165].

There are several models affecting N-module subunits. Mutations in the *NDUFV1* homolog *nuo-1* result in hallmark features of CI deficiency, including reduced CI activity, lactic acidosis and increased oxidative stress sensitivity [114]. Models targeting the *C. elegans NDUFS4* homolog *lpd-5* and the *NDUFS1* homolog *nuo-5* generated by RNAi-mediated depletion recapitulate biochemical (decreased mitochondrial respiration), cellular (induction of mitochondrial stress responses), behavioural (neuromuscular defects) and developmental (slow growth) characteristics of the corresponding human disease [118]. These models allowed the identification of a neuroligin-mediated cholinergic defects underlying neuronal defects induced by mitochondrial CI dysfunction and to screen an array of natural compounds for potential therapeutic benefit [118], which have been recently validated in newly developed CRISPR/Cas9-mediated lines introducing patient-associated *NDUFS1/nuo-5* mutations (*author unpublished communication*). Further on, homozygous *NDUFS4*/*lpd-5* knock out worms with CRISPR/Cas9-mediated 354 bp deletion resulting in a 103 amino acid deletion and frameshift have unravelled a protective effect on worm growth and development, which however cannot be replicated in *NDUFS3/nuo-2*, *NDUFS1/nuo-5*, *NDUFS7/nduf-7* mutants [119]. Other defects in Q-module subunits produce similar phenotypes as variants of the N-module. Mutations in the *NDUFA6* homolog *nuo-3* similarly confer hypoxia-mediated protection [119], while disruption of the *NDUFS2* homolog *gas-1* leads to impaired mitochondrial function and reduced lifespan, somehow mirroring human CI deficiency [133]. Other genetically engineered *C. elegans* models targeting *NDUFV1*/*nuo*-*1* and *NDUFA10* homolog *nuo-4* have also been applied in therapeutic discovery. While the *NDUFV1*/*nuo-1* model did not fully recapitulate human pathophysiology, a *NDUFA10/nuo-4* model proved effective for demonstrating beneficial effects of hydrogen sulphide donor AP39 on mitochondrial function [166].

#### *Drosophilamelanogaster*

The fruit fly *Drosophila melanogaster* represents another well-established invertebrate model organism for mitochondrial disease research. Its short life cycle and fully sequenced, highly conserved genome, make *Drosophila* a cost- and time-efficient system for preclinical research of CI deficiency [167]. Accordingly, a range of *Drosophila* models for CI deficiency have been generated, most commonly by RNAi-mediated depletion of CI subunits or engineered using the GAL4/UAS system [167].

Several models targeting N- and Q-module subunits have been described. Foriel et al. generated RNAi-mediated models targeting the *NDUFS4* homolog *ND-18*, *NDUFS7* homolog *ND-20* and *NDUFV1* homolog *ND-51*, subunits that are all associated with severe, early-onset mitochondrial disease. Across multiple driver lines, these models showed decreased CI activity, secondary alteration in other respiratory complexes, shortened lifespan and impaired development, with *NDUFS7/ND-20* producing the mildest and *NDUFV1/ND-51* the most severe phenotype [115].

*Drosophila* models also capture the phenotypic variability observed in human CI deficiency to a certain extent. Indeed, variable RNAi-mediated depletion of *NDUFS1* homolog *ND-75* demonstrated a clear correlation between depletion and disease severity, affecting lifespan, behaviour, mitochondrial morphology, metabolism and gamma-aminobutyric acid (GABA) levels, hinting on potential neuronal dysfunction [120].

In addition to RNAi-mediated approaches, genetically engineered *Drosophila* models have been generated to functionally validate disease-associated variants. A GAL4/UAS-mediated model carrying a pathogenic variant of *NDUFS7* homolog *ND-20* was originally developed to assess genetic variants found in dogs with LS enabling improved genetic testing and supporting the use of spontaneous large mammal models for research of CI deficiency [168].

Models targeting the *NDUFS8* homolog *ND-23* have been used to investigate LS and MELAS related pathomechanisms [139]. RNAi- and GAL4/UAS-mediated *NDUFS8/ND-23* knockdown induces neuronal loss, retinal degeneration and disrupted lipid metabolism with lipid droplet accumulation in glial cells, recapitulating key features of CI deficiency. In response, enhancing the neuronal glucose uptake partially rescued neurodegeneration, whereas glial pathology persisted, which highlights the potential role for lipid homeostasis in CI-associated disease [139]. Beyond nuclear encoded subunits, *Drosophila* models harbouring mutations in mtDNA encoded CI subunit *ND2* have also been established [141, 169]. These models are affected in their lifespan and behaviour and exhibit seizures, neurodegeneration and decreased CI activity, linked to impaired proton pumping [141]. Similar to findings to findings in *NDUFS8*/*ND-23* knockdown models, *ND2* mutants show defects in fatty acid and lipid storage. Pharmacological inhibition of TOR signalling by rapamycin rescued lifespan and lipid storage defects independently of autophagy, underscoring metabolic modulations as a potential therapeutic strategy in CI deficiency [169].

#### Zebrafish *Danio rerio*

The zebrafish *Danio rerio* is a small fresh water vertebrate widely used to model neuropathologies not only due to high genetic conservation but also thanks to the highly conserved structure and functions of its nervous system with humans [170–172]. Accordingly, *Danio rerio* has become a valuable system for studying mitochondrial gene mutations underlying human neurological disease [173]. For many years, only a single *Danio rerio* model for CI-associated mitochondrial dysfunction was available, based on the knockout of CI assembly factor *ndufaf7* rather than a structural CI subunit [174]. *ndufaf7* knockouts exhibit impaired CI assembly, decreased ATP levels and behavioural and cardiac defects, partially recapitulating clinical features of human CI deficiency [174]. More recently, Mitchell et al. successfully generated a *ndufs2* knockout model, thereby expanding the *Danio rerio* CI disease model repertoire. *ndufs2*^−/−^ mutants show markedly reduced CI activity with associated morphological and metabolic defects, including dysregulated lipid metabolism and hepatomegaly, a phenotype also reported in LS patients [135]. Notably, these defects can be partially rescued by folate supplementation [135].

#### Mice

The development of mammalian models, particularly in mice (*Mus musculus*) has greatly advanced the understanding of CI deficiency. The most commonly used *in vivo* model is the systemic *Ndufs4* knockout mouse, in which the homozygous deletion of exon 2 causes a severe phenotype close to LS at molecular, structural, and behavioural levels [123, 124, 175–177]. As homozygous models for other mutations are often non-viable, heterozygous models are used to reproduce milder clinical phenotypes [178]. Recent studies in *Ndufs4* knockout mice revealed an intact blood–brain barrier, highlighting the need for central nervous system targeted delivery of gene or pharmacological therapies [179]. Cell type-specific analysis using embryonic stem cells, neurons and astrocytes derived from these mice demonstrated impaired CI activity, assembly and ATP production with increased ROS in a cell-dependent manner, highlighting tissue-specific vulnerability [180]. Conditional and tissue-specific *Ndufs4* knockouts further clarified CI-related pathomechanisms. Cardiac-specific *Ndufs4* knockouts revealed that a reduced NAD+/NADH ratio promotes protein acetylation and heart failure [181]. Neuron- and glia-specific models showed that degeneration of striatal neurons underlies motor deficits in CI deficiency [182], while progressive glial activation contributes to neuronal loss and ultimately mortality [183]. The *Ndufs4* model has also facilitated therapeutic development, showing that cholesterol biosynthesis stimulation improves motor function and survival [184], and hypoxia exposure prevents or partially reverses neurological symptoms, supporting pharmacological strategies aimed at normalizing brain hyperoxia [185, 186].

A second systemic CI model, the *Ndufs6* knockout mouse, exhibits tissue-specific CI deficiency with a pronounced defect in the heart resulting in cardiomyopathy and heart failure [132]. In contrast to the primarily neurological phenotype of *Ndufs4* knockouts [123, 175], *Ndufs6* knockout mice model cardiac clinical features of human CI deficiency and moreover implicate CI deficiency as a risk factor for chronic renal disease [187].

Mouse models also enable mechanistic studies using primary cells. Microglia-specific CI deficiency modelled by *Ndufs2* knockout mice, causes alteration in lifespan, behaviour and synaptic function, accompanied by reduced respiration and phagocytic capacity in microglia, indicating a critical role for CI in preventing neuroinflammation during development [134]. The neuron-specific *Ndufs3* knockout mouse model has proven equally valuable [137, 188]. Loss of *Ndufs3* in neurons decreases CI activity, alters brain metabolism, and impairs motor coordination. Compensatory increases in glycolysis motivated evaluation of metformin, which delayed symptom onset [137]. More recently, adeno-associated virus-mediated *Ndufs3* gene replacement therapy restored CI function, lifespan, and behaviour, although clinical translation so far is limited and may be further complicated by a narrow therapeutic window [188].

Disease models in mouse also exist for mtDNA encoded CI subunits. *ND6* mutant mice carrying a pathogenic point mutation exhibit defects in CI assembly and activity, increased ROS, and optic neuropathy resembling LHON, including age-related retinal decline which is reflective of human CI deficiency [153].

Additionally, an X-chromosome linked *Ndufa1* knock-in mouse generated by homologous recombination revealed sex-dependent disease predispositions, with divergent neurodegeneration and survival patterns between males and females indicating a potential gender bias in CI-deficiency [189].

### Human-derived *in vitro* models

Multicellular model organisms are essential to recapitulate *in vivo* phenotypic and behavioural aspects of CI deficiency-associated diseases and to unravel pathomechanistic aspects for targeted therapeutic strategies. Nonetheless, *in vitro* cellular disease models are still of high priority for biochemistry, metabolic and cell-specific studies especially aimed at validating therapeutic approaches in human relevant systems. Even though relatively little genetic models have been developed in the past, considering the heterogenous clinical presentation of CI deficiency associated diseases, various *in vitro* systems have been utilized. These are critically summarized below (Table 1; Figs. 2 and 3).

#### Human fibroblasts

Human patient-derived fibroblasts are not only widely used to investigate the biochemical mechanisms of CI deficiency, but also support diagnostic evaluation. Improved cellular assays are indeed critical for accelerating diagnosis and enabling early interventions, which may ameliorate disease progression in CI deficiency-associated diseases such as LS, MELAS or LHON, despite the lack of curative therapies [184–186].

Patient-derived fibroblast obtained from skin biopsies carry the complete individual genetic background and therefore represent valuable case-specific disease models, potentially useful for personalized therapies, particularly given the limited availability of genetic CI deficiency models. Fibroblasts harbouring pathogenic variants in nuclear-encoded CI subunits *NDUFS1*, *NDUFS2*, *NDUFS4*, *NDUFS7*, *NDUFS8*,* NDUFV1* and *NDUFA12* as well as mtDNA encoded subunits *ND1*, *ND3*, *ND4* and *ND5* have been extensively characterised. Frequently investigated parameters of mitochondrial function including CI enzymatic activity and assembly or ROS production have been described in Sect.  3.1–3.3 [47, 116, 136].

Patient-derived fibroblasts with CI deficiency commonly exhibit decreased CI enzyme activity, impaired CI protein assembly and increased levels of ROS, as described in cases affecting subunits *NDUFS1*,* NDUFS2*,* NDUFS7*,* NDUFS8* or *NDUFV1* [47, 136]. However, mitochondrial dysfunction varies substantially between and within affected subunits and although patient-derived fibroblasts often recapitulate cellular disease-relevant phenotypes, their diagnostic and prognostic utility is limited. For instance, pathogenic variants of *NDUFS4* in fibroblasts display drastic cellular impairment such as variable alterations in mitochondrial membrane potential, ROS levels, Ca^2+^ signalling, ATP homeostasis, morphology and CI assembly [66, 78], yet cohort studies revealed no correlation between residual CI activity and clinical severity or age of onset [125].

Despite these limitations, patient-derived fibroblasts have been instrumental in elucidating disease mechanism and evaluating therapeutic strategies. CI deficiency disrupts ATP production and mitochondrial Ca^2+^ homeostasis [126] and similar to findings in mouse models for CI deficiency, NAD^+^-boosting approaches improve mitochondrial parameters in fibroblasts carrying pathogenic variants of *NDUFS1* [181, 190]. Consistent with adaptive metabolic compensation, CI-deficient fibroblasts upregulate glycolysis and show increased cell death when substituting glucose with galactose, which can be rescued by NAD^+^ and pyruvate supplementation [191]. Fibroblasts harbouring mtDNA-encoded pathogenic variants in subunits *ND1*,* ND3* or *ND5* have further revealed dysregulated Ca^2+^ signalling, increased mitophagy and oxidative stress, with beneficial effects observed upon modulation of pro-oxidative pathways [140, 148].

Recent studies have demonstrated the feasibility of gene-based therapeutic strategies in fibroblasts with mtDNA encoded CI mutations. Import of ND3 protein into mitochondria partially restored CI function, supporting ongoing efforts to develop allotopic expression-based gene therapies for mitochondrial disease [192, 193]. Additional approaches include coculture of patient-derived fibroblasts with mesenchymal cells, which exert improvement on ROS production, redox homeostasis and mitochondrial function across models with nuclear and mtDNA encoded mutations, depending on their paracrine properties by secreting microvesicles and exosomes independent of mitochondrial transfer [194, 195]. Other patient-derived models, such as lymphoblastoid cell lines, are available but are less commonly used [110].

#### Pluripotent stem cells

Selecting disease models that reflect key clinical features, which are relevant for human phenotypes of CI deficiency is critical. However, the predominant neurological involvement of CI deficiency is often not adequately captured in patient-derived fibroblasts. Human induced pluripotent stem cells (hiPSCs) overcome this limitation, as they can be differentiated into neurons, cardiomyocytes or organoids, thereby enabling more accurate modelling of human CI deficiency. hiPSCs are commonly generated by reprogramming somatic cells, such as fibroblasts or peripheral blood mononuclear cells, using retroviral transduction [117, 196].

Over the past decade, numerous hiPSC-based models for CI deficiency have been established, targeting both nuclear encoded CI subunits *NDUFS1*, *NDUFS4* and *NDUVF1* and mtDNA encoded *ND3*, *ND4* and *ND*. Patient-specific hiPSCs have been generated from individuals with LS carrying pathogenic variants in N-module subunit *NDUFV1* [117] or *NDUFS1* [121], providing variant-resolved models for CI deficiency. *NDUFS4*, an accessory subunit involved in CI assembly and stability, is a particularly well-studied target. hiPSCs derived from patients harbouring pathogenic variants of *NDUFS4* or generated via CRISPR/Cas9-mediated knockout exhibit decreased CI activity, decreased NDUFS4 protein levels and a lowered NAD^+^/NADH ratio, consistent with findings in *Ndufs4* knockout mice [128, 181, 191]. Pharmacological modulation of NAD^+^ metabolism, including ß-Lapachone treatment, partially restored redox balance and decreased oxidative stress in these models [128].

Differentiation of *NDUFS4* mutant hiPSCs into astrocytes [130] or into neural progenitors, neurons and brain organoids has been successful and a valuable addition to the repertoire of disease models. It revealed defects in mitochondrial and neuronal morphogenesis, accompanied by increased oxidative stress and heightened susceptibility to excitotoxicity [127, 129]. Importantly, organoid-based models implicated microglial activation and pro-inflammatory signalling in CI deficiency-associated neurodegeneration, in agreement with observations from *Ndufs4* knockout mice and supporting immunomodulating approaches as potential therapeutic strategies [127]. In parallel, differentiation of *NDUFS4* knockout hiPSCs into mixed neuronal and cardiomyocyte populations demonstrated pronounced cell type-specific vulnerability. Neuronal apoptosis was driven by NAD^+^ depletion and protein hyperacetylation, whereas cardiomyocytes displayed altered inward sodium currents, suggesting a mechanistic link to arrhythmogenic phenotypes observed in CI [131].

hiPSCs models have also substantially advanced the study of CI deficiency caused by mtDNA encoded mutations. hiPSCs harbouring pathogenic variants of *ND4* generated from LHON patient-derived cells and differentiated into retinal ganglion cells exhibited impaired differentiation, altered morphology and functional defects, closely recapitulating disease-relevant phenotypes and providing further evidence for synergistic effects between pathogenic variants [146]. hiPSCs harbouring pathogenic *ND3* variants associated with MELAS/LS overlap revealed metabolomic alterations only at high mutant loads and predominantly in the undifferentiated state, suggesting that cellular energy demand critically influences phenotypic expression and may underlie stress-induced metabolic decompensation in patients [143]. Similarly, LS patient-derived hiPSCs carrying *ND3* or *ND5* mutations displayed variant-specific defects in mitochondrial function, membrane potential and dynamics, with no uniform genotype-phenotype correlation [144]. *ND5* mutant hiPSCs-derived neurons and cardiomyocytes further demonstrated impaired CI activity and mitochondrial function as well as disturbed Ca^2+^ homeostasis, highlighting calcium dysregulation as a central feature of CI deficiency [149–151]. Notably, differentiation studies revealed *ND5* mutant-load-dependent effects on the lineage specification and on cellular vulnerability in iPSCs-derived cardiomyocytes. High heteroplasmy levels biased differentiation towards neuroectodermal lineages, whereas mutant loads preferentially impaired the cellular function. These findings provide a mechanistic explanation for tissue-specific involvement and variable penetrance observed in CI deficiency-associated diseases, often causing involvement of the brain but not always of the heart [152].

#### Human continuous cell lines

Human continuous cell lines provide a simple, inexpensive and scalable *in vitro* system to model CI deficiency-associated disease and are particularly valuable for large-scale and mechanistic studies. However, relatively few genetically defined CI deficiency models have been established in immortalized human cell lines to date. Mutations in nuclear encoded CI subunits have predominantly been introduced into human embryonic kidney HEK293T cells, which enable validation of findings from animal models in a human cellular context and facilitate detailed molecular analysis. A prominent example is the comprehensive study by Stroud et al., in which HEK293T knockout lines for all accessory CI subunits were generated and analysed by proteomics, demonstrating their essential role in CI [23]. Using CRISPR/Cas9, additional HEK293T knockout models targeting *NDUFS4*, *NDUFS2* and *NDUFS7* have been established [131, 138]. These models recapitulate key clinical features of CI deficiency observed in other systems, including decreased mitochondrial membrane potential, increased ROS production, altered protein acetylation and impaired depolarizing Na^+^ currents, the latter being particularly pronounced in *NDUFS2* mutant cells and consistent with observations in *NDUFS4* mutant hiPSC-derived cardiomyocytes [131]. N*DUFS7* knockout cells further exhibit reduced proliferation and increased susceptibility to oxidative stress-induced apoptosis, which can be partially mitigated by enhanced cystine import and glutathione biosynthesis [138].

When modeling CI deficiency associated with mtDNA encoded subunits, cytoplasmic hybrid (cybrid) cell lines are the method of choice. Cybrids are generated by fusing a nuclear donor cell line, such as 143B-derived cells, with patient-derived cytoplasts carrying pathogenic mtDNA variants, enabling the isolation of mtDNA effects on cellular function [197]. Cybrid models have been instrumental in identifying pathogenic mtDNA variants associated with LHON and LS [95]. Cybrids carrying *ND1*, *ND2*, *ND4* or *ND5* mutations display impaired CI activity and assembly, decreased NADPH levels, increased apoptosis and altered mitophagy, and also have revealed synergistic effects of mtDNA mutations contributing to severity of disease phenotype [110, 142, 147], as described in other model systems as well. Notably, combined non-pathogenic mtDNA variants in *ND4*, *ND4L* and *ND6* can also reduce CI activity and trigger to this a study using cybrid cell lines for *ND4*, *ND4L* and *ND6* has revealed that even nonpathogenic variants of mitochondrial DNA can in combination lead to reduced CI activity and trigger LHON phenotypes [113]. Moreover, cybrid models carrying LHON-associated *ND6* mutations, have facilitated optimization of the allotopic expression strategies, with successful rescue of CI defects upon *ND6* transgene delivery [154, 155].

### Comparison and considerations of available disease models

Taken together all information on available CI deficiency models, the advantages and limitations of each must be evaluated in the context of their intended application (Fig. 3). A central consideration in model selection is the pronounced phenotypic heterogeneity of CI deficiency, which requires choosing a system that recapitulates the clinical features of interest best, such as neurological, cardiac or metabolic involvement. Inevitably, this selection involves trade-offs between biological complexity, translational relevance and experimental accessibility.

The first selection at the highest level, is the choice between CI deficiency models in *in vivo* model organisms and in *in vitro* cellular models. *In vivo* models include non-mammalian organisms such as *C. elegans*, *Drosophila* and *Danio rerio*, as well as mammalian mouse models. A major advantage of *in vivo* systems is their ability to capture disease processes at the level of the whole organism, integrating tissue-specific effects with inter-tissue communications with possible repercussion on developmental, systemic and behavioral phenotypes that cannot be reproduced in isolated cell cultures. Established CI deficiency models in *C. elegans* and *Drosophila* are particularly attractive due to their fully sequenced genomes, which show a high degree of conservation with humans, including mitochondrial pathways and the ETC [114, 167]. Their accessibility is increased by their short life cycles, enabling lifespan, behavioral and stress-response analyses in a time- and cost-efficient manner, and by powerful genetic tools such as RNAi, CRISPR/Cas9 and GAL4/UAS systems, facilitating genotype-specific manipulation as well as genotype-phenotype correlation studies [198]. Due to their simple yet functionally conserved nervous systems, neurobehavioral assays in these models allow identification of relevant diseases suppressors. The optical transparency of *C. elegans* further enables various *in vivo* analysis. *C. elegans* very short generation time of 3–5, days, its microscopic size and its simple and inexpensive growth conditions contribute to make this model organism very favorable for mitochondrial stress response studies, to research pharmacological targets and even for large-scale or high-throughputs drug screening [118, 133, 199]. However, studies with *C. elegans* as a model are set back by the simple nervous system compared to humans, limiting the modelling of complex, human-relevant pathological features (e.g., brain stem lesions). *Drosophila* models showing higher homology to humans than *C. elegans*, have proven to replicate some tissue- and organ-specific aspects of CI deficiency, particularly neurological phenotypes [120, 139], although their throughput for compound or genetic suppressors screening is more limited compared to *C. elegans* [198].

Owing to their fast generation time, genetic accessibility and range of available models, both invertebrate systems currently offer greater experimental flexibility than *Danio rerio*, even though *Danio rerio* provide the advantage of a vertebrate context, making its anatomy and physiology more similar to humans, with a highly conserved nervous and cardiovascular systems and the optical transparency of the larvae facilitate *in vivo* analysis [170, 171, 198]. However, their lower throughput and the relatively limited availability of CI-specific genetic models have constrained their broader use in CI deficiency research [135, 174]. A common limitation of all non-mammalian models is their restricted physiological similarity to humans, including differences in the neuronal system and metabolic regulation which limits their ability to fully recapitulate complex tissue- and organ-specific pathologies. Consequently, findings from these systems require careful interpretation and validation in more human-relevant models.

Mouse models occupy a critical intermediate position, combining mammalian physiology with the ability to model both nuclear and mtDNA encoded CI defects in systemic or tissue-specific contexts. They enable the study of more complex organ-level pathomechanisms, disease progression and therapeutic interventions in a preclinical setting, albeit often only partially reproducing human clinical phenotypes and showing limited viability in severe models [124, 175, 176, 178, 200]. Their experimental accessibility is further constrained by high costs, long generation times and ethical considerations that restrict experimental throughput [198]. These limitations underscore the need to complement *in vivo* studies in non-mammalian and mammalian organisms with human-derived *in vitro* systems, which provide a human-relevant and experimentally tractable platform while reducing the use of extensive animal experimentation. Human *in vitro* models represent an essential component in CI deficiency research, each offering distinct strengths while also presenting important limitations. Patient-derived fibroblasts retain the complete patient-specific genetic background and are widely accessible, making them valuable for biochemical profiling, diagnostic assay development and initial therapeutic screening, mainly in clinical context [184–186]. However, their limited relevance for predominantly neurological clinical features in CI deficiency, combined with poor genotype–phenotype correlation and restricted predictive value for disease severity, limits their utility for mechanistic and translational studies [47, 125]. hiPSCs overcome several of these limitations by enabling differentiation into disease-relevant cell types, such as cardiomyocytes or astrocytes, neurons and brain organoids, thereby allowing investigation of tissue-specific vulnerability, heteroplasmy thresholds and patient- or variant-specific phenotypes, whilst retaining the patient’s genetic background in cases of patient-derived hiPSCs [121, 127, 130]. hiPSCs provide a powerful platform for modelling neurological, retinal and cardiac CI disorders and for developing gene- and metabolism-based therapies, but their accessibility is limited by technical complexity, regarding handling, quality control and high cost. Especially the hiPSC-based modelling of mtDNA encoded variants remains challenging due to mitochondrial segregation, heteroplasmy and challenging derivation of isogenic hiPSC lines [196, 201]. Incomplete maturation can further hinder the research of CI deficiency, due to reliance on glycolysis instead of OXPHOS for energy generation in hiPSCs [196]. Continuous human cell lines and cybrid models offer a more accessible complementary approach, as they are simple, scalable and highly reproducible, making them well suited for mechanistic studies, molecular pathway analyses and variant pathogenicity testing [23, 131, 138]. Cybrid systems, in particular, conveniently allow the isolation and functional assessment of mtDNA variants in a controlled nuclear background [110, 142, 197]. However, genetic models in immortalized cell lines are currently only available for a limited number of CI subunits and are predominantly generated in HEK293T cells, a human cell line with limited translational relevance for predominantly neurodevelopmental CI deficiency-associated diseases [23, 131, 138]. Moreover, continuous cell line-based models can potentially be confounded by cancer-associated metabolic alterations and lack the ability to replicate complex cellular, tissue or organ-specific interactions, thereby restricting their physiological relevance. Collectively, although available *in vitro* systems have substantially advanced understanding of CI deficiency, no single model fully recapitulates the complexity of human CI deficiency-associated disease. Consequently, model selection should always be guided by the specific research question and experimental objective, with careful consideration of respective strengths and limitations of each system. A summary of the main advantages, limitations and optimal applications of the available CI deficiency models is provided in Figs. 2 and 3, intended to support informed model selection. Importantly, research of CI deficiency cannot rely on a single experimental system but benefits most from a complementary use of *in vivo* and *in vitro* systems, which can be achieved mainly via large collaborative efforts [118, 119, 127, 131]. At the same time, the limitations of current disease models reveal a persistent gap in physiologically relevant and scalable disease models, underscoring the need for more advanced disease models on all experimental levels. Future progress will depend on next-generation, integrative modelling strategies that combine complementary *in vivo* and *in vitro* systems, with model selection tailored to the specific research objective and clinical context.

## Conclusions

Mitochondrial CI is the largest and one of the most critical enzymes of the respiratory chain, playing a central role in mitochondrial function and ATP production. Defects in CI are the most common cause of mitochondrial disorders, including LS, LHON and MELAS. Despite the identification of numerous mutations across multiple CI subunits, there remains a lack of clear correlation between specific genetic variants and clinical manifestations. This genetic and phenotypic heterogeneity continues to pose significant challenges for understanding disease mechanisms and for the development of effective therapies.

Over the past decades, a broad spectrum of disease models has substantially advanced the field. These range from invertebrate organisms such as *C. elegans* and *Drosophila* to vertebrates like *Danio rerio* and finally mammalian systems including mice, patient-derived fibroblasts, hiPSC-derived cells and organoids, and continuous human cell lines. Each of these systems has provided important insights into CI assembly, mitochondrial defects and pathomechanisms underlying diseases as well as potential therapeutic strategies. For instance, the synergistic effects of pathogenic mtDNA variants and the comparatively clear correlation between genotype and phenotype observed in LHON, the only CI deficiency-associated disorder with such a defined correlation, have successfully been reproduced and investigated across multiple disease models [110, 112, 142, 146, 147]. However, no single model fully captures the systemic, tissue-specific, and genetic complexity of human mitochondrial disease, underscoring the need for complementary, multisystemic-model approaches. A major remaining gap lies in the availability of cost-efficient, highly scalable mammalian models suitable for high-throughput genetic and pharmacological screening. While currently available invertebrate models and immortalized cell lines offer scalability and experimental tractability, their physiological relevance to human disease is limited. Conversely, mammalian *in vivo* models and hiPSC-derived systems provide higher translational value but remain technically demanding, costly, and low in throughput. Furthermore, the use of mammalian *in vivo* models is subject to strict ethical considerations and should be reserved for questions that cannot be adequately addressed using alternative systems. Developing scalable, human-relevant platforms, such as advanced engineered cell lines as well as a standardized hiPSC-based systems could therefore be essential for the research of CI deficiency, more extensively facilitating systematic multi-omics analyses and high throughput therapeutic screening. Another persistent challenge concerns mtDNA encoded CI variants. These mutations are difficult to model due to heteroplasmy and the lack of robust genome-editing tools for mtDNA [196, 201]. Although cybrid systems enable the study of isolated mtDNA effects, they lack tissue context and physiological complexity. Emerging hiPSC-based approaches hold promise, but stable modelling of heteroplasmic mtDNA mutations remains technically challenging and requires further methodological advances [196].

Recent work across multiple model systems has also begun to highlight previously underexplored pathomechanisms, including alterations in fatty acid storage, lipid metabolism, and cholesterol biosynthesis [135, 139, 169, 184]. These findings suggest that CI deficiency affects cellular metabolism more broadly than previously appreciated and may open new therapeutic approaches. In parallel, interventions such as controlled hypoxia or metabolic modulation have shown beneficial effects in several experimental models, indicating that targeting systemic metabolic states may represent a promising treatment strategy. More advanced CI deficiency disease models such as *Ndufs3* knockout mice [188] or cybrid cell lines [154, 155] have also enabled the development of gene therapy approaches targeting both nuclear and, increasingly, mtDNA and raise the prospect of future curative treatments of CI deficiency-associated diseases [201–203]. Future efforts will likely focus on the development of humanized and hybrid model systems that combine scalability with physiological relevance. Such approaches may include platforms linking high-throughput invertebrate and human *in vitro* models with organoid systems and finally *in vivo* validation. These next-generation models will be crucial for unraveling the complexity of CI deficiency-associated diseases and translating mechanistic insights into targeted, effective treatments tailored to specific genetic backgrounds and clinical phenotypes.

## Supplementary Information

Below is the link to the electronic supplementary material.

Supplementary Material 1

## Data Availability

Not applicable.

## References

[1] Moser CC, Farid TA, Chobot SE, Dutton PL (2006) Electron tunneling chains of mitochondria. Biochimica et Biophysica Acta (BBA) - Bioenergetics. 1757(9):1096–109. 10.1016/j.bbabio.2006.04.015 10.1016/j.bbabio.2006.04.015 16780790

[2] Kwong JQ, Beal MF, Manfredi G (2006) The role of mitochondria in inherited neurodegenerative diseases. J Neurochem 97(6):1659–1675. 10.1111/j.1471-4159.2006.03990.x 16805775 10.1111/j.1471-4159.2006.03990.x

[3] Distelmaier F, Koopman WJ, van den Heuvel LP, Rodenburg RJ, Mayatepek E, Willems PH et al (2009) Mitochondrial complex I deficiency: from organelle dysfunction to clinical disease. Brain 132(Pt 4):833–842. 10.1093/brain/awp058 19336460 10.1093/brain/awp058

[4] Henke MT, Prigione A, Schuelke M (2024) Disease models of Leigh syndrome: From yeast to organoids. J Inherit Metab Dis 47(6):1292–1321. 10.1002/jimd.12804 39385390 10.1002/jimd.12804 PMC11586605

[5] Vercellino I, Sazanov LA (2022) The assembly, regulation and function of the mitochondrial respiratory chain. Nat Rev Mol Cell Biol 23(2):141–161. 10.1038/s41580-021-00415-0 34621061 10.1038/s41580-021-00415-0

[6] Hirst J, Mitochondrial Complex I (2013) Annu Rev Biochem 82(1):551–. 10.1146/annurev-biochem-070511-103700. 75 23527692 10.1146/annurev-biochem-070511-103700

[7] Belevich I, Verkhovsky MI, Wikström M (2006) Proton-coupled electron transfer drives the proton pump of cytochrome c oxidase. Nature 440(7085):829–832. 10.1038/nature04619 16598262 10.1038/nature04619

[8] Mitchell P (2011) Chemiosmotic coupling in oxidative and photosynthetic phosphorylation. Biochimica et Biophysica Acta (BBA). - Bioenergetics 1807(12):1507–1538. 10.1016/j.bbabio.2011.09.018 10.1016/j.bbabio.2011.09.018 22082452

[9] Protasoni M, Zeviani M (2021) Mitochondrial Structure and Bioenergetics in Normal and Disease Conditions. Int J Mol Sci. 10.3390/ijms22020586 33435522 10.3390/ijms22020586 PMC7827222

[10] Liu H, Li H, Yao X, Yan X, Peng R (2025) Environmental nanoplastics induce mitochondrial dysfunction: A review of cellular mechanisms and associated diseases. Environ Pollut 382:126695. 10.1016/j.envpol.2025.126695 40550382 10.1016/j.envpol.2025.126695

[11] Akinrinde A, Sanchez LA, Maglioni S, Ventura N (2026) *Caenorhabditis elegans* as a Model System for Environmental Mitotoxicants. Ann Rev Phramacol Toxicol 66(28):1–24. *in press*10.1146/annurev-pharmtox-062124-012254 41270292

[12] Fassone E, Rahman S (2012) Complex I deficiency: clinical features, biochemistry and molecular genetics. J Med Genet 49(9):578–590. 10.1136/jmedgenet-2012-101159 22972949 10.1136/jmedgenet-2012-101159

[13] Sharma LK, Lu J, Bai Y (2009) Mitochondrial respiratory complex I: structure, function and implication in human diseases. Curr Med Chem 16(10):1266–1277. 10.2174/092986709787846578 19355884 10.2174/092986709787846578 PMC4706149

[14] Hatefi Y, Haavik AG, Griffiths DE (1962) Studies on the electron transfer system. XL. Preparation and properties of mitochondrial DPNH-coenzyme Q reductase. J Biol Chem 237:1676–1680 13905327

[15] Heron C, Smith S, Ragan CI (1979) An analysis of the polypeptide composition of bovine heart mitochondrial NADH-ubiquinone oxidoreductase by two-dimensional polyacrylamide-gel electrophoresis. Biochem J 181(2):435–443. 10.1042/bj1810435 496892 10.1042/bj1810435 PMC1161175

[16] Fiedorczuk K, Sazanov LA (2018) Mammalian Mitochondrial Complex I Structure and Disease-Causing Mutations. Trends Cell Biol 28(10):835–. 10.1016/j.tcb.2018.06.006. 67 30055843 10.1016/j.tcb.2018.06.006

[17] Baradaran R, Berrisford JM, Minhas GS, Sazanov LA (2013) Crystal structure of the entire respiratory complex I. Nature 494(7438):443–448. 10.1038/nature11871 23417064 10.1038/nature11871 PMC3672946

[18] Efremov RG, Sazanov LA (2011) Structure of the membrane domain of respiratory complex I. Nature 476(7361):414–420. 10.1038/nature10330 21822288 10.1038/nature10330

[19] Carroll J, Fearnley IM, Shannon RJ, Hirst J, Walker JE (2003) Analysis of the subunit composition of complex I from bovine heart mitochondria. Mol Cell Proteom 2(2):117–126. 10.1074/mcp.M300014-MCP200 10.1074/mcp.M300014-MCP200 12644575

[20] Carroll J, Fearnley IM, Skehel JM, Shannon RJ, Hirst J, Walker JE (2006) Bovine Complex I Is a Complex of 45 Different Subunits *. J Biol Chem 281(43):32724–32727. 10.1074/jbc.M607135200 16950771 10.1074/jbc.M607135200

[21] Balsa E, Marco R, Perales-Clemente E, Szklarczyk R, Calvo E, Landázuri Manuel O et al (2012) NDUFA4 Is a Subunit of Complex IV of the Mammalian Electron Transport Chain. Cell Metabol 16(3):378–386. 10.1016/j.cmet.2012.07.015 10.1016/j.cmet.2012.07.015 22902835

[22] Wirth C, Brandt U, Hunte C, Zickermann V (2016) Structure and function of mitochondrial complex I. Biochimica et Biophysica Acta (BBA) -. Bioenergetics 1857(7):902–914. https://doi.org/https://doi.10.1016/j.bbabio.2016.02.013 10.1016/j.bbabio.2016.02.013 26921811

[23] Stroud DA, Surgenor EE, Formosa LE, Reljic B, Frazier AE, Dibley MG et al (2016) Accessory subunits are integral for assembly and function of human mitochondrial complex I. Nature 538(7623):123–126. 10.1038/nature19754 27626371 10.1038/nature19754

[24] Kahlhöfer F, Gansen M, Zickermann V (2021) Accessory Subunits of the Matrix Arm of Mitochondrial Complex I with a Focus on Subunit NDUFS4 and Its Role in Complex I Function and Assembly. Life (Basel) 11(5). 10.3390/life11050455 10.3390/life11050455 PMC8161149 34069703

[25] Lazarou M, Thorburn DR, Ryan MT, McKenzie M (2009) Assembly of mitochondrial complex I and defects in disease. Biochim Biophys Acta 1793(1):78–88. 10.1016/j.bbamcr.2008.04.015 18501715 10.1016/j.bbamcr.2008.04.015

[26] Chomyn A, Mariottini P, Cleeter MW, Ragan CI, Matsuno-Yagi A, Hatefi Y et al (1985) Six unidentified reading frames of human mitochondrial DNA encode components of the respiratory-chain NADH dehydrogenase. Nature 314(6012):592–597. 10.1038/314592a0 3921850 10.1038/314592a0

[27] Carroll J, Ding S, Fearnley IM, Walker JE (2013) Post-translational modifications near the quinone binding site of mammalian complex I. J Biol Chem 288(34):24799–24808. 10.1074/jbc.M113.488106 23836892 10.1074/jbc.M113.488106 PMC3750175

[28] Vartak RS, Semwal MK, Bai Y (2014) An update on complex I assembly: the assembly of players. J Bioenerg Biomembr 46(4):323–328. 10.1007/s10863-014-9564-x 25030182 10.1007/s10863-014-9564-x PMC4412850

[29] Parey K, Wirth C, Vonck J, Zickermann V (2020) Respiratory complex I — structure, mechanism and evolution. Curr Opin Struct Biol 63:1–9. 10.1016/j.sbi.2020.01.004 32058886 10.1016/j.sbi.2020.01.004

[30] Schulte M, Frick K, Gnandt E, Jurkovic S, Burschel S, Labatzke R et al (2019) A mechanism to prevent production of reactive oxygen species by Escherichia coli respiratory complex I. Nat Commun 10(1):2551. 10.1038/s41467-019-10429-0 31186428 10.1038/s41467-019-10429-0 PMC6560083

[31] Signorile A, De Rasmo D (2023) Mitochondrial Complex I, a Possible Sensible Site of cAMP Pathway in Aging. Antioxidants 12(2):221 36829783 10.3390/antiox12020221 PMC9951957

[32] Guan S, Zhao L, Peng R (2022) Mitochondrial Respiratory Chain Supercomplexes: From Structure to Function. Int J Mol Sci 23(22). 10.3390/ijms232213880 10.3390/ijms232213880 PMC9696846 36430359

[33] Kampjut D, Sazanov LA (2020) The coupling mechanism of mammalian respiratory complex I. Science 370(6516). 10.1126/science.abc4209 10.1126/science.abc4209 32972993

[34] Rahman S, Blok RB, Dahl HH, Danks DM, Kirby DM, Chow CW et al (1996) Leigh syndrome: clinical features and biochemical and DNA abnormalities. Ann Neurol 39(3):343–351. 10.1002/ana.410390311 8602753 10.1002/ana.410390311

[35] Loeffen JL, Smeitink JA, Trijbels JM, Janssen AJ, Triepels RH, Sengers RC et al (2000) Isolated complex I deficiency in children: clinical, biochemical and genetic aspects. Hum Mutat 15(2):123–134. 10.1002/(SICI)1098-1004(200002)15:2<123::AID-HUMU1>3.0.CO;2-P 10.1002/(SICI)1098-1004(200002)15:2<123::AID-HUMU1>3.0.CO;2-P 10649489

[36] Bugiani M, Invernizzi F, Alberio S, Briem E, Lamantea E, Carrara F et al (2004) Clinical and molecular findings in children with complex I deficiency. Biochim Biophys Acta 1659(2–3):136–147. 10.1016/j.bbabio.2004.09.006 15576045 10.1016/j.bbabio.2004.09.006

[37] Meyerson C, Van Stavern G, McClelland C (2015) Leber hereditary optic neuropathy: current perspectives. Clin Ophthalmol 9:1165–1176. 10.2147/opth.S62021 26170609 10.2147/OPTH.S62021 PMC4492634

[38] Finsterer J (2008) Leigh and Leigh-Like Syndrome in Children and Adults. Pediatr Neurol 39(4):223–. 10.1016/j.pediatrneurol.2008.07.013. 35 18805359 10.1016/j.pediatrneurol.2008.07.013

[39] Leigh D (1951) Subacute necrotizing encephalomyelopathy in an infant. J Neurol Neurosurg Psychiatry 14(3):216–221. 10.1136/jnnp.14.3.216 14874135 10.1136/jnnp.14.3.216 PMC499520

[40] Wijburg FA, Wanders RJA, van Lie Peters EM, Vos GD, Loggers HG, Bolhuis PA et al (1991) NADH:Q1 oxidoreductase deficiency without lactic acidosis in a patient with leigh syndrome: Implications for the diagnosis of inborn errors of the respiratory chain. J Inherit Metab Dis 14(3):297–300. 10.1007/BF01811686 1770777 10.1007/BF01811686

[41] Zhang Y, Yang YL, Sun F, Cai X, Qian N, Yuan Y et al (2007) Clinical and molecular survey in 124 Chinese patients with Leigh or Leigh-like syndrome. J Inherit Metab Dis 30(2):265. 10.1007/s10545-006-0481-y 17323145 10.1007/s10545-006-0481-y

[42] Henry C, Patel N, Shaffer W, Murphy L, Park J, Spieler B (2017) Mitochondrial Encephalomyopathy With Lactic Acidosis and Stroke-Like Episodes-MELAS Syndrome. Ochsner J 17(3):296–301 29026367 PMC5625994

[43] Kaufmann P, Engelstad K, Wei Y, Kulikova R, Oskoui M, Sproule DM et al (2011) Natural history of MELAS associated with mitochondrial DNA m.3243A > G genotype. Neurology 77(22):1965–1971. 10.1212/WNL.0b013e31823a0c7f 22094475 10.1212/WNL.0b013e31823a0c7f PMC3235358

[44] Hirano M, Ricci E, Richard Koenigsberger M, Defendini R, Pavlakis SG, DeVivo DC et al (1992) MELAS: An original case and clinical criteria for diagnosis. Neuromuscul Disord 2(2):125–135. 10.1016/0960-8966(92)90045-8 1422200 10.1016/0960-8966(92)90045-8

[45] Newman NJ (2005) Hereditary optic neuropathies: from the mitochondria to the optic nerve. Am J Ophthalmol 140(3):517–. 10.1016/j.ajo.2005.03.017. 23 16083845 10.1016/j.ajo.2005.03.017

[46] Yu-Wai-Man P, Griffiths PG, Hudson G, Chinnery PF (2009) Inherited mitochondrial optic neuropathies. J Med Genet 46(3):145–158. 10.1136/jmg.2007.054270 19001017 10.1136/jmg.2007.054270 PMC2643051

[47] Bénit P, Chretien D, Kadhom N, de Lonlay-Debeney P, Cormier-Daire V, Cabral A et al (2001) Large-scale deletion and point mutations of the nuclear NDUFV1 and NDUFS1 genes in mitochondrial complex I deficiency. Am J Hum Genet 68(6):1344–1352. 10.1086/320603 11349233 10.1086/320603 PMC1226121

[48] Calvo SE, Tucker EJ, Compton AG, Kirby DM, Crawford G, Burtt NP et al (2010) High-throughput, pooled sequencing identifies mutations in NUBPL and FOXRED1 in human complex I deficiency. Nat Genet 42(10):851–858. 10.1038/ng.659 20818383 10.1038/ng.659 PMC2977978

[49] Kiss S, Christodoulou J, Thorburn DR, Freeman JL, Kornberg AJ, Mandelstam S et al (2023) A cryptic pathogenic variant identified by RNA-seq in a patient with normal complex I activity in muscle and transient magnetic resonance imaging changes. Am J Med Genet Part A 191(6):1599–1606. 10.1002/ajmg.a.63170 36896486 10.1002/ajmg.a.63170

[50] Schuelke M, Smeitink J, Mariman E, Loeffen J, Plecko B, Trijbels F et al (1999) Mutant NDUFV1 subunit of mitochondrial complex I causes leukodystrophy and myoclonic epilepsy. Nat Genet 21(3):260–261. 10.1038/6772 10080174 10.1038/6772

[51] Zanette V, Valle Dd, Telles BA, Robinson AJ, Monteiro V, Santos MLSF et al (2021) *NDUFV1* mutations in complex I deficiency: Case reports and review of symptoms. Genet Mol Biology 44(4):e20210149. 10.1590/1678-4685-GMB-2021-0149.eCollection2021 10.1590/1678-4685-GMB-2021-0149 PMC8607527 34807224

[52] Breningstall GN, Shoffner J, Patterson RJ (2008) Siblings with leukoencephalopathy. Semin Pediatr Neurol 15(4):212–215. 10.1016/j.spen.2008.10.013 19073330 10.1016/j.spen.2008.10.013

[53] Becker N, Sharma A, Gosse M, Kubat B, Conway KS (2022) The neuropathologic findings in a case of progressive cavitating leukoencephalopathy due to NDUFV1 pathogenic variants. Acta Neuropathol Commun 10(1):142. 10.1186/s40478-022-01445-1 36163075 10.1186/s40478-022-01445-1 PMC9511743

[54] Bénit P, Beugnot R, Chretien D, Giurgea I, De Lonlay-Debeney P, Issartel JP et al (2003) Mutant NDUFV2 subunit of mitochondrial complex I causes early onset hypertrophic cardiomyopathy and encephalopathy. Hum Mutat 21(6):582–586. 10.1002/humu.10225 12754703 10.1002/humu.10225

[55] Kishita Y, Shimura M, Kohda M, Fushimi T, Nitta KR, Yatsuka Y et al (2021) Genome sequencing and RNA-seq analyses of mitochondrial complex I deficiency revealed Alu insertion-mediated deletion in NDUFV2. Hum Mutat 42(11):1422–1428. 10.1002/humu.24274 34405929 10.1002/humu.24274

[56] Pagniez-Mammeri H, Lombes A, Brivet M, Ogier-de Baulny H, Landrieu P, Legrand A et al (2009) Rapid screening for nuclear genes mutations in isolated respiratory chain complex I defects. Mol Genet Metab 96(4):196–200. 10.1016/j.ymgme.2008.12.003 19167255 10.1016/j.ymgme.2008.12.003

[57] Bénit P, Steffann J, Lebon S, Chretien D, Kadhom N, de Lonlay P et al (2003) Genotyping microsatellite DNA markers at putative disease loci in inbred/multiplex families with respiratory chain complex I deficiency allows rapid identification of a novel nonsense mutation (IVS1nt -1) in the NDUFS4 gene in Leigh syndrome. Hum Genet 112(5–6):563–566. 10.1007/s00439-002-0884-2 12616398 10.1007/s00439-002-0884-2

[58] Hoefs SJ, Skjeldal OH, Rodenburg RJ, Nedregaard B, van Kaauwen EP, Spiekerkötter U et al (2010) Novel mutations in the NDUFS1 gene cause low residual activities in human complex I deficiencies. Mol Genet Metab 100(3):251–256. 10.1016/j.ymgme.2010.03.015 20382551 10.1016/j.ymgme.2010.03.015

[59] Ferreira M, Torraco A, Rizza T, Fattori F, Meschini MC, Castana C et al (2011) Progressive cavitating leukoencephalopathy associated with respiratory chain complex I deficiency and a novel mutation in NDUFS1. Neurogenetics 12(1):9–17. 10.1007/s10048-010-0265-2 21203893 10.1007/s10048-010-0265-2

[60] Iuso A, Scacco S, Piccoli C, Bellomo F, Petruzzella V, Trentadue R et al (2006) Dysfunctions of Cellular Oxidative Metabolism in Patients with Mutations in the NDUFS1 and NDUFS4 Genes of Complex I*. J Biol Chem 281(15):10374–10380. 10.1074/jbc.M513387200 16478720 10.1074/jbc.M513387200

[61] Martín MA, Blázquez A, Gutierrez-Solana LG, Fernández-Moreira D, Briones P, Andreu AL et al (2005) Leigh Syndrome Associated With Mitochondrial Complex I Deficiency Due to a Novel Mutation in the NDUFS1 Gene. Arch Neurol 62(4):659–661. 10.1001/archneur.62.4.659 15824269 10.1001/archneur.62.4.659

[62] Men L, Feng J, Huang W, Xu M, Zhao X, Sun R et al (2022) Lip cyanosis as the first symptom of Leigh syndrome associated with mitochondrial complex I deficiency due to a compound heterozygous NDUFS1 mutation: A case report. Med (Baltim) 101(34):e30303. 10.1097/md.0000000000030303 10.1097/MD.0000000000030303 PMC9410648 36042640

[63] Pagniez-Mammeri H, Landrieu P, Legrand A, Slama A (2010) Leukoencephalopathy with vanishing white matter caused by compound heterozygous mutations in mitochondrial complex I NDUFS1 subunit. Mol Genet Metab 101(2):297–298. 10.1016/j.ymgme.2010.07.005 20797884 10.1016/j.ymgme.2010.07.005

[64] Tuppen HAL, Hogan VE, He L, Blakely EL, Worgan L, Al-Dosary M et al (2010) The p.M292T NDUFS2 mutation causes complex I-deficient Leigh syndrome in multiple families. Brain 133(10):2952–2963. 10.1093/brain/awq232 20819849 10.1093/brain/awq232 PMC2947428

[65] Anderson SL, Chung WK, Frezzo J, Papp JC, Ekstein J, DiMauro S et al (2008) A novel mutation in NDUFS4 causes Leigh syndrome in an Ashkenazi Jewish family. J Inherit Metab Dis 31(Suppl 2):S461–S467. 10.1007/s10545-008-1049-9 19107570 10.1007/s10545-008-1049-9

[66] Budde SM, van den Heuvel LP, Janssen AJ, Smeets RJ, Buskens CA, DeMeirleir L et al (2000) Combined enzymatic complex I and III deficiency associated with mutations in the nuclear encoded NDUFS4 gene. Biochem Biophys Res Commun 275(1):63–68. 10.1006/bbrc.2000.3257 10944442 10.1006/bbrc.2000.3257

[67] Kirby DM, Salemi R, Sugiana C, Ohtake A, Parry L, Bell KM et al (2004) NDUFS6 mutations are a novel cause of lethal neonatal mitochondrial complex I deficiency. J Clin Investig 114(6):837–845. 10.1172/JCI20683 15372108 10.1172/JCI20683 PMC516258

[68] Li Y, Zhang Y, Jiang G, Wang Y, He C, Zhao X et al (2022) Case report: novel mutations of NDUFS6 and NHLRC2 genes potentially cause the quick postnatal death of a Chinese Hani minority neonate with mitochondrial complex I deficiency and FINCA syndrome. Med (Baltim) 101(27):e29239. 10.1097/md.0000000000029239 10.1097/MD.0000000000029239 PMC9259100 35801790

[69] Rouzier C, Chaussenot A, Fragaki K, Serre V, Ait-El-Mkadem S, Richelme C et al (2019) NDUFS6 related Leigh syndrome: a case report and review of the literature. J Hum Genet 64(7):637–645. 10.1038/s10038-019-0594-4 30948790 10.1038/s10038-019-0594-4

[70] Spiegel R, Shaag A, Mandel H, Reich D, Penyakov M, Hujeirat Y et al (2009) Mutated NDUFS6 is the cause of fatal neonatal lactic acidemia in Caucasus Jews. Eur J Hum Genet 17(9):1200–1203. 10.1038/ejhg.2009.24 19259137 10.1038/ejhg.2009.24 PMC2986593

[71] Hoefs SJ, Dieteren CE, Distelmaier F, Janssen RJ, Epplen A, Swarts HG et al (2008) NDUFA2 complex I mutation leads to Leigh disease. Am J Hum Genet 82(6):1306–1315. 10.1016/j.ajhg.2008.05.007 18513682 10.1016/j.ajhg.2008.05.007 PMC2427319

[72] Magrinelli F, Cali E, Braga VL, Yis U, Tomoum H, Shamseldin H et al (2022) Biallelic Loss-of-Function NDUFA12 Variants Cause a Wide Phenotypic Spectrum from Leigh/Leigh-Like Syndrome to Isolated Optic Atrophy. Mov Disord Clin Pract 9(2):218–228. 10.1002/mdc3.13398 35141356 10.1002/mdc3.13398 PMC8810437

[73] Ostergaard E, Rodenburg RJ, van den Brand M, Thomsen LL, Duno M, Batbayli M et al (2011) Respiratory chain complex I deficiency due to NDUFA12 mutations as a new cause of Leigh syndrome. J Med Genet 48(11):737–740. 10.1136/jmg.2011.088856 21617257 10.1136/jmg.2011.088856

[74] Perrier S, Gauquelin L, Tétreault M, Tran LT, Webb N, Srour M et al (2018) Recessive mutations in NDUFA2 cause mitochondrial leukoencephalopathy. Clin Genet 93(2):396–400. 10.1111/cge.13126 28857146 10.1111/cge.13126

[75] Yatsuka Y, Kishita Y, Formosa LE, Shimura M, Nozaki F, Fujii T et al (2020) A homozygous variant in NDUFA8 is associated with developmental delay, microcephaly, and epilepsy due to mitochondrial complex I deficiency. Clin Genet 98(2):155–165. 10.1111/cge.13773 32385911 10.1111/cge.13773

[76] Zafeiriou DI, Rodenburg RJ, Scheffer H, van den Heuvel LP, Pouwels PJ, Ververi A et al (2008) MR spectroscopy and serial magnetic resonance imaging in a patient with mitochondrial cystic leukoencephalopathy due to complex I deficiency and NDUFV1 mutations and mild clinical course. Neuropediatrics 39(3):172–175. 10.1055/s-0028-1093336 18991197 10.1055/s-0028-1093336

[77] Haddad S, Salloum E, Silan A, Kalecioğlu G, Abdulnour M, Haddad S et al (2025) Mitochondrial complex I deficiency in a 4-year-old boy due to compound heterozygous NDUFV1 mutation: a case report of a new pathogenic variant. Oxf Med Case Rep 2025(4):omae166. 10.1093/omcr/omae166 10.1093/omcr/omae166 PMC11979451 40207266

[78] González-Quintana A, Trujillo-Tiebas MJ, Fernández-Perrone AL, Blázquez A, Lucia A, Morán M et al (2020) Uniparental isodisomy as a cause of mitochondrial complex I respiratory chain disorder due to a novel splicing NDUFS4 mutation. Mol Genet Metab 131(3):341–348. 10.1016/j.ymgme.2020.10.008 33093004 10.1016/j.ymgme.2020.10.008

[79] Loeffen J, Elpeleg O, Smeitink J, Smeets R, Stöckler-Ipsiroglu S, Mandel H et al (2001) Mutations in the complex I NDUFS2 gene of patients with cardiomyopathy and encephalomyopathy. Ann Neurol 49(2):195–201. 10.1002/1531-8249(20010201)49:2<195::aid-ana39>3.0.co;2-m 11220739 10.1002/1531-8249(20010201)49:2<195::aid-ana39>3.0.co;2-m

[80] Marin SE, Mesterman R, Robinson B, Rodenburg RJ, Smeitink J, Tarnopolsky MA (2013) Leigh syndrome associated with mitochondrial complex I deficiency due to novel mutations In NDUFV1 and NDUFS2. Gene 516(1):162–167. 10.1016/j.gene.2012.12.024 23266820 10.1016/j.gene.2012.12.024

[81] Rubrecht A, Clapp W, Shenoy A (2020) Liver Pathology in Mitochondrial Complex I Deficiency from Bi-Allelic Mutations in NDUFS2: A Report of Findings at Autopsy. Fetal Pediatr Pathol 39(3):259–262. 10.1080/15513815.2019.1651800 31411514 10.1080/15513815.2019.1651800

[82] Bénit P, Slama A, Cartault F, Giurgea I, Chretien D, Lebon S et al (2004) Mutant NDUFS3 subunit of mitochondrial complex I causes Leigh syndrome. J Med Genet 41(1):14–17. 10.1136/jmg.2003.014316 14729820 10.1136/jmg.2003.014316 PMC1757256

[83] Johnstone T, Wang J, Ross D, Balanda N, Huang Y, Godfrey R et al (2020) Biallelic variants in two complex I genes cause abnormal splicing defects in probands with mild Leigh syndrome. Mol Genet Metab 131(1–2):98–106. 10.1016/j.ymgme.2020.09.008 33097395 10.1016/j.ymgme.2020.09.008 PMC7749052

[84] Lou X, Shi H, Wen S, Li Y, Wei X, Xie J et al (2018) A Novel NDUFS3 mutation in a Chinese patient with severe Leigh syndrome. J Hum Genet 63(12):1269–1272. 10.1038/s10038-018-0505-0 30140060 10.1038/s10038-018-0505-0

[85] Triepels RH, van den Heuvel LP, Loeffen JL, Buskens CA, Smeets RJ, Rubio Gozalbo ME et al (1999) Leigh syndrome associated with a mutation in the NDUFS7 (PSST) nuclear encoded subunit of complex I. Ann Neurol 45(6):787–790. 10.1002/1531-8249(199906)45:6<787::aid-ana13>3.0.co;2-6 10360771 10.1002/1531-8249(199906)45:6<787::aid-ana13>3.0.co;2-6

[86] Loeffen J, Smeitink J, Triepels R, Smeets R, Schuelke M, Sengers R et al (1998) The first nuclear-encoded complex I mutation in a patient with Leigh syndrome. Am J Hum Genet 63(6):1598–1608. 10.1086/302154 9837812 10.1086/302154 PMC1377631

[87] Gowda VK, Bylappa AY, Kinhal U, Srinivasan VM, Vamyanmane DK (2023) Mitochondrial Complex I Deficiency Masquerading as Stroke-Like Episode Clinically and as Alexander Disease Radiologically Following Chicken Pox. Ann Indian Acad Neurol 26(6):977–979. 10.4103/aian.aian_339_23 38229652 10.4103/aian.aian_339_23 PMC10789425

[88] Alston CL, Heidler J, Dibley MG, Kremer LS, Taylor LS, Fratter C et al (2018) Bi-allelic Mutations in NDUFA6 Establish Its Role in Early-Onset Isolated Mitochondrial Complex I Deficiency. Am J Hum Genet 103(4):592–601. 10.1016/j.ajhg.2018.08.013 30245030 10.1016/j.ajhg.2018.08.013 PMC6174280

[89] Simon DK, Friedman J, Breakefield XO, Jankovic J, Brin MF, Provias J et al (2003) A heteroplasmic mitochondrial complex I gene mutation in adult-onset dystonia. Neurogenetics 4(4):199–205. 10.1007/s10048-003-0150-3 12756609 10.1007/s10048-003-0150-3

[90] Kirby DM, McFarland R, Ohtake A, Dunning C, Ryan MT, Wilson C et al (2004) Mutations of the mitochondrial ND1 gene as a cause of MELAS. J Med Genet 41(10):784–789. 10.1136/jmg.2004.020537 15466014 10.1136/jmg.2004.020537 PMC1735602

[91] Howell N, Bindoff LA, McCullough DA, Kubacka I, Poulton J, Mackey D et al (1991) Leber hereditary optic neuropathy: identification of the same mitochondrial ND1 mutation in six pedigrees. Am J Hum Genet 49(5):939–950 1928099 PMC1683233

[92] Danhelovska T, Kolarova H, Zeman J, Hansikova H, Vaneckova M, Lambert L et al (2020) Multisystem mitochondrial diseases due to mutations in mtDNA-encoded subunits of complex I. BMC Pediatr 20(1):41. 10.1186/s12887-020-1912-x 31996177 10.1186/s12887-020-1912-x PMC6988306

[93] Lou X, Zhou Y, Liu Z, Xie Y, Zhang L, Zhao S et al (2023) De novo frameshift variant in MT-ND1 causes a mitochondrial complex I deficiency associated with MELAS syndrome. Gene 860:147229. 10.1016/j.gene.2023.147229 36717040 10.1016/j.gene.2023.147229

[94] Johns DR, Berman J (1991) Alternative, simultaneous complex I mitochondrial DNA mutations in Leber’s hereditary optic neuropathy. Biochem Biophys Res Commun 174(3):1324–1330. 10.1016/0006-291x(91)91567-v 1900003 10.1016/0006-291x(91)91567-v

[95] Xu M, Kopajtich R, Elstner M, Li H, Liu Z, Wang J et al (2022) Identification of a novel m.3955G > A variant in MT-ND1 associated with Leigh syndrome. Mitochondrion 62:13–23. 10.1016/j.mito.2021.10.002 34656796 10.1016/j.mito.2021.10.002

[96] McFarland R, Kirby DM, Fowler KJ, Ohtake A, Ryan MT, Amor DJ et al (2004) De novo mutations in the mitochondrial ND3 gene as a cause of infantile mitochondrial encephalopathy and complex I deficiency. Ann Neurol 55(1):58–64. 10.1002/ana.10787 14705112 10.1002/ana.10787

[97] Tolomeo D, Rubegni A, Severino M, Pochiero F, Bruno C, Cassandrini D et al (2019) Clinical and neuroimaging features of the m.10197G > A mtDNA mutation: New case reports and expansion of the phenotype variability. J Neurol Sci 399:69–75. 10.1016/j.jns.2019.02.010 30776730 10.1016/j.jns.2019.02.010

[98] Lin Y, Xu X, Zhao D, Liu F, Luo Y, Du J et al (2020) A novel m.11406 T > A mutation in mitochondrial ND4 gene causes MELAS syndrome. Mitochondrion 54:57–64. 10.1016/j.mito.2020.06.011 32659360 10.1016/j.mito.2020.06.011

[99] Barone V, La Morgia C, Caporali L, Fiorini C, Carbonelli M, Gramegna LL et al (2022) Case Report: Optic Atrophy and Nephropathy With m.13513G > A/MT-ND5 mtDNA Pathogenic Variant. Front Genet 13:887696. 10.3389/fgene.2022.887696 35719398 10.3389/fgene.2022.887696 PMC9204033

[100] Zhang AM, Jia X, Guo X, Zhang Q, Yao Y-G (2012) Mitochondrial DNA mutation m.10680G > A is associated with Leber hereditary optic neuropathy in Chinese patients. J Translational Med 10(1):43. 10.1186/1479-5876-10-43 10.1186/1479-5876-10-43 PMC3372436 22400981

[101] Brown MD, Voljavec AS, Lott MT, MacDonald I, Wallace DC (1992) Leber’s hereditary optic neuropathy: a model for mitochondrial neurodegenerative diseases. Faseb j 6(10):2791–2799. 10.1096/fasebj.6.10.1634041 1634041 10.1096/fasebj.6.10.1634041

[102] Ravn K, Wibrand F, Hansen FJ, Horn N, Rosenberg T, Schwartz M (2001) An mtDNA mutation, 14453G–>A, in the NADH dehydrogenase subunit 6 associated with severe MELAS syndrome. Eur J Hum Genet 9(10):805–809. 10.1038/sj.ejhg.5200712 11781695 10.1038/sj.ejhg.5200712

[103] Ugalde C, Triepels RH, Coenen MJH, Van Den Heuvel LP, Smeets R, Uusimaa J et al (2003) Impaired complex I assembly in a Leigh syndrome patient with a novel missense mutation in the ND6 gene. Ann Neurol 54(5):665–669. 10.1002/ana.10734 14595656 10.1002/ana.10734

[104] Vandeputte J, Mattias VH, Caroline VC, Sara S, Elfride DB, LB P et al (2021) Mild Leber hereditary optic neuropathy (LHON) in a Western European family due to the rare Asian m.14502T > C variant in the MT-ND6 gene. Ophthalmic Genet 42(4):440–445. 10.1080/13816810.2021.1913611 33858285 10.1080/13816810.2021.1913611

[105] Ng YS, Thompson K, Loher D, Hopton S, Falkous G, Hardy SA et al (2020) Novel MT-ND Gene Variants Causing Adult-Onset Mitochondrial Disease and Isolated Complex I Deficiency. Front Genet 11:24. 10.3389/fgene.2020.00024 32158465 10.3389/fgene.2020.00024 PMC7052259

[106] Alahmad A, Nasca A, Heidler J, Thompson K, Oláhová M, Legati A et al (2020) Bi‐allelic pathogenic variants in < i>NDUFC2 cause early‐onset Leigh syndrome and stalled biogenesis of complex I. EMBO Mol Med 12(11):e12619. 10.15252/emmm.202012619 32969598 10.15252/emmm.202012619 PMC7645371

[107] Kaiyrzhanov R, Thompson K, Efthymiou S, Mukushev A, Zharylkassyn A, Prasad C et al (2025) Biallelic NDUFA13 variants lead to a neurodevelopmental phenotype with gradual neurological impairment. Brain Commun 7(1):fcae453. 10.1093/braincomms/fcae453 39963288 10.1093/braincomms/fcae453 PMC11832047

[108] Alila OF, Rebai EM, Tabebi M, Tej A, Chamkha I, Tlili A et al (2016) Whole mitochondrial genome analysis in two families with dilated mitochondrial cardiomyopathy: detection of mutations in MT-ND2 and MT-TL1 genes. Mitochondrial DNA DNA Mapp Seq Anal 27(4):2873–2880. 10.3109/19401736.2015.1060417 10.3109/19401736.2015.1060417 26258512

[109] Jiang Z, Teng L, Zhang S, Ding Y (2021) Mitochondrial ND1 T4216C and ND2 C5178A mutations are associated with maternally transmitted diabetes mellitus. Mitochondrial DNA DNA Mapp Seq Anal 32(2):59–65. 10.1080/24701394.2020.1856101 10.1080/24701394.2020.1856101 33284036

[110] Ji Y, Zhang J, Lu Y, Yi Q, Chen M, Xie S et al (2020) Complex I mutations synergize to worsen the phenotypic expression of Leber’s hereditary optic neuropathy. J Biol Chem 295(38):13224–13238. 10.1074/jbc.RA120.014603 32723871 10.1074/jbc.RA120.014603 PMC7504918

[111] Nissanka N, Moraes CT (2020) Mitochondrial DNA heteroplasmy in disease and targeted nuclease-based therapeutic approaches. EMBO Rep 21(3):e49612. 10.15252/embr.201949612 32073748 10.15252/embr.201949612 PMC7054667

[112] Mkaouar-Rebai E, Ammar M, Sfaihi L, Alila-Fersi O, Maalej M, Felhi R et al (2021) Mitochondrial disease patients with novel ND4 12058A > C and ND1 m.3911A > G variations: implications for a role in the phenotype following a bioinformatic investigation. Mol Biol Rep 48(5):4373–4382. 10.1007/s11033-021-06452-4 34089464 10.1007/s11033-021-06452-4

[113] Caporali L, Iommarini L, La Morgia C, Olivieri A, Achilli A, Maresca A et al (2018) Peculiar combinations of individually non-pathogenic missense mitochondrial DNA variants cause low penetrance Leber’s hereditary optic neuropathy. PLoS Genet 14(2):e1007210. 10.1371/journal.pgen.1007210 29444077 10.1371/journal.pgen.1007210 PMC5828459

[114] Grad LI, Lemire BD (2004) Mitochondrial complex I mutations in *Caenorhabditis elegans* produce cytochrome c oxidase deficiency, oxidative stress and vitamin-responsive lactic acidosis. Hum Mol Genet 13(3):303–314. 10.1093/hmg/ddh027 14662656 10.1093/hmg/ddh027

[115] Foriel S, Renkema GH, Lasarzewski Y, Berkhout J, Rodenburg RJ, Smeitink JAM et al (2019) A *Drosophila**Mitochondrial* Complex I Deficiency Phenotype Array. Front Genet 10:245. 10.3389/fgene.2019.00245 30972103 10.3389/fgene.2019.00245 PMC6445954

[116] Koopman WJ, Verkaart S, van Emst-de Vries SE, Grefte S, Smeitink JA, Nijtmans LG et al (2008) Mitigation of NADH: ubiquinone oxidoreductase deficiency by chronic Trolox treatment. Biochim Biophys Acta 1777(7–8):853–859. 10.1016/j.bbabio.2008.03.028 18435906 10.1016/j.bbabio.2008.03.028

[117] Sequiera GL, Rockman-Greenberg C, Dhingra S (2020) Induced pluripotent stem cell line UOMi002-A from a patient with Leigh syndrome with compound heterozygous mutations in the NDUFV1 gene. Stem Cell Res 48:101964. 10.1016/j.scr.2020.101964 32871395 10.1016/j.scr.2020.101964

[118] Maglioni S, Schiavi A, Melcher M, Brinkmann V, Luo Z, Laromaine A et al (2022) Neuroligin-mediated neurodevelopmental defects are induced by mitochondrial dysfunction and prevented by lutein in C. elegans. Nat Commun 13(1):2620. 10.1038/s41467-022-29972-4 35551180 10.1038/s41467-022-29972-4 PMC9098500

[119] Meisel JD, Miranda M, Skinner OS, Wiesenthal PP, Wellner SM, Jourdain AA et al (2024) Hypoxia and intra-complex genetic suppressors rescue complex I mutants by a shared mechanism. Cell 187(3):659–75e18. 10.1016/j.cell.2023.12.010 38215760 10.1016/j.cell.2023.12.010 PMC10919891

[120] Granat L, Knorr DY, Ranson DC, Chakrabarty RP, Chandel NS, Bateman JM (2024) A Drosophila model of mitochondrial disease phenotypic heterogeneity. Biol Open 13(2). 10.1242/bio.060278 10.1242/bio.060278 PMC10924217 38304969

[121] Valente O, Dobner J, Ramachandran H, Hildebrandt B, Distelmaier F, Ventura N et al (2022) Generation of an induced pluripotent stem cell line (IUFi002-A) from a Leigh syndrome patient carrying mutations in the NDUFS1 gene. Stem Cell Res 65:102971. 10.1016/j.scr.2022.102971 36403546 10.1016/j.scr.2022.102971

[122] Abrahams JP, Leslie AG, Lutter R, Walker JE (1994) Structure at 2.8 A resolution of F1-ATPase from bovine heart mitochondria. Nature 370(6491):621–628. 10.1038/370621a0 8065448 10.1038/370621a0

[123] Kruse SE, Watt WC, Marcinek DJ, Kapur RP, Schenkman KA, Palmiter RD (2008) Mice with mitochondrial complex I deficiency develop a fatal encephalomyopathy. Cell Metab 7(4):312–320. 10.1016/j.cmet.2008.02.004 18396137 10.1016/j.cmet.2008.02.004 PMC2593686

[124] Yin Z, Agip AA, Bridges HR, Hirst J (2024) Structural insights into respiratory complex I deficiency and assembly from the mitochondrial disease-related ndufs4(-/-) mouse. Embo j 43(2):225–249. 10.1038/s44318-023-00001-4 38177503 10.1038/s44318-023-00001-4 PMC10897435

[125] Koene S, Rodenburg RJ, van der Knaap MS, Willemsen MA, Sperl W, Laugel V et al (2012) Natural disease course and genotype-phenotype correlations in Complex I deficiency caused by nuclear gene defects: what we learned from 130 cases. J Inherit Metab Dis 35(5):737–747. 10.1007/s10545-012-9492-z 22644603 10.1007/s10545-012-9492-z PMC3432203

[126] Willems PH, Valsecchi F, Distelmaier F, Verkaart S, Visch HJ, Smeitink JA et al (2008) Mitochondrial Ca2 + homeostasis in human NADH:ubiquinone oxidoreductase deficiency. Cell Calcium 44(1):123–133. 10.1016/j.ceca.2008.01.002 18295330 10.1016/j.ceca.2008.01.002

[127] Daneshgar N, Leidinger MR, Le S, Hefti M, Prigione A, Dai DF (2022) Activated microglia and neuroinflammation as a pathogenic mechanism in Leigh syndrome. Front Neurosci 16:1068498. 10.3389/fnins.2022.1068498 36741056 10.3389/fnins.2022.1068498 PMC9889986

[128] Goolab S, Terburgh K, du Plessis C, Scholefield J, Louw R (2025) CRISPR-Cas9 mediated knockout of NDUFS4 in human iPSCs: A model for mitochondrial complex I deficiency. Biochim Biophys Acta Mol Basis Dis 1871(2):167569. 10.1016/j.bbadis.2024.167569 39547516 10.1016/j.bbadis.2024.167569

[129] Inak G, Rybak-Wolf A, Lisowski P, Pentimalli TM, Jüttner R, Glažar P et al (2021) Defective metabolic programming impairs early neuronal morphogenesis in neural cultures and an organoid model of Leigh syndrome. Nat Commun 12(1):1929. 10.1038/s41467-021-22117-z 33771987 10.1038/s41467-021-22117-z PMC7997884

[130] Sonsalla G, Malpartida AB, Riedemann T, Gusic M, Rusha E, Bulli G et al (2024) Direct neuronal reprogramming of NDUFS4 patient cells identifies the unfolded protein response as a novel general reprogramming hurdle. Neuron 112(7):1117–32e9. 10.1016/j.neuron.2023.12.020 38266647 10.1016/j.neuron.2023.12.020 PMC10994141

[131] Yoon J-Y, Daneshgar N, Chu Y, Chen B, Hefti M, Vikram A et al (2022) Metabolic rescue ameliorates mitochondrial encephalo-cardiomyopathy in murine and human iPSC models of Leigh syndrome. Clin Translational Med 12(7):e954. 10.1002/ctm2.954 10.1002/ctm2.954 PMC9309541 35872650

[132] Ke BX, Pepe S, Grubb DR, Komen JC, Laskowski A, Rodda FA et al (2012) Tissue-specific splicing of an Ndufs6 gene-trap insertion generates a mitochondrial complex I deficiency-specific cardiomyopathy. Proc Natl Acad Sci U S A 109(16):6165–6170. 10.1073/pnas.1113987109 22474353 10.1073/pnas.1113987109 PMC3341001

[133] McCormack S, Polyak E, Ostrovsky J, Dingley SD, Rao M, Kwon YJ et al (2015) Pharmacologic targeting of sirtuin and PPAR signaling improves longevity and mitochondrial physiology in respiratory chain complex I mutant *Caenorhabditis elegans*. Mitochondrion 22:45–59. 10.1016/j.mito.2015.02.005 25744875 10.1016/j.mito.2015.02.005 PMC4447550

[134] Mora-Romero B, Capelo-Carrasco N, Pérez-Moreno JJ, Alvarez-Vergara MI, Trujillo-Estrada L, Romero-Molina C et al (2024) Microglia mitochondrial complex I deficiency during development induces glial dysfunction and early lethality. Nat Metabolism 6(8):1479–1491. 10.1038/s42255-024-01081-0 10.1038/s42255-024-01081-0 39048800

[135] Mitchell DV, Iadarola DM, Mathew ND, Keith K, Seiler C, Yu S et al (2025) ndufs2(-/-) zebrafish have impaired survival, neuromuscular activity, morphology, and one-carbon metabolism treatable with folic acid. bioRxiv. 10.1101/2025.07.16.66492941573838

[136] Ehinger JK, Piel S, Ford R, Karlsson M, Sjövall F, Frostner E et al (2016) Cell-permeable succinate prodrugs bypass mitochondrial complex I deficiency. Nat Commun 7:12317. 10.1038/ncomms12317 27502960 10.1038/ncomms12317 PMC4980488

[137] Peralta S, Pinto M, Arguello T, Garcia S, Diaz F, Moraes CT (2020) Metformin delays neurological symptom onset in a mouse model of neuronal complex I deficiency. JCI Insight 5(21). 10.1172/jci.insight.141183 10.1172/jci.insight.141183 PMC7710273 33148885

[138] Chen J, Gao L (2024) SLC7A11-mediated cystine import protects against NDUFS7 deficiency-induced cell death in HEK293T cells. Biochem Biophys Res Commun 723:150178. 10.1016/j.bbrc.2024.150178 38823363 10.1016/j.bbrc.2024.150178

[139] Cabirol-Pol MJ, Khalil B, Rival T, Faivre-Sarrailh C, Besson MT (2018) Glial lipid droplets and neurodegeneration in a Drosophila model of complex I deficiency. Glia 66(4):874–888. 10.1002/glia.23290 29285794 10.1002/glia.23290

[140] Wojtala A, Karkucinska-Wieckowska A, Sardao VA, Szczepanowska J, Kowalski P, Pronicki M et al (2017) Modulation of mitochondrial dysfunction-related oxidative stress in fibroblasts of patients with Leigh syndrome by inhibition of prooxidative p66Shc pathway. Mitochondrion 37:62–79. 10.1016/j.mito.2017.07.002 28739512 10.1016/j.mito.2017.07.002

[141] Burman JL, Itsara LS, Kayser EB, Suthammarak W, Wang AM, Kaeberlein M et al (2014) A Drosophila model of mitochondrial disease caused by a complex I mutation that uncouples proton pumping from electron transfer. Dis Model Mech 7(10):1165–1174. 10.1242/dmm.015321 25085991 10.1242/dmm.015321 PMC4174527

[142] Del Prado L, Jaraíz-Rodríguez M, Agro M, Zamora-Dorta M, Azpiazu N, Calleja M et al (2024) Compensatory activity of the PC-ME1 metabolic axis underlies differential sensitivity to mitochondrial complex I inhibition. Nat Commun 15(1):8682. 10.1038/s41467-024-52968-1 39375345 10.1038/s41467-024-52968-1 PMC11458614

[143] Hattori T, Hamazaki T, Kudo S, Shintaku H (2016) Metabolic Signature of MELAS/Leigh Overlap Syndrome in Patient-specific Induced Pluripotent Stem Cells Model. Osaka City Med J 62(2):69–76 30721581

[144] Meshrkey F, Cabrera Ayuso A, Rao RR, Iyer S (2021) Quantitative analysis of mitochondrial morphologies in human induced pluripotent stem cells for Leigh syndrome. Stem Cell Res 57:102572. 10.1016/j.scr.2021.102572 34662843 10.1016/j.scr.2021.102572 PMC10332439

[145] Brown MD, Starikovskaya E, Derbeneva O, Hosseini S, Allen JC, Mikhailovskaya IE et al (2002) The role of mtDNA background in disease expression: a new primary LHON mutation associated with Western Eurasian haplogroup J. Hum Genet 110(2):130–138. 10.1007/s00439-001-0660-8 11935318 10.1007/s00439-001-0660-8

[146] Nie Z, Wang C, Chen J, Ji Y, Zhang H, Zhao F et al (2023) Abnormal morphology and function in retinal ganglion cells derived from patients-specific iPSCs generated from individuals with Leber’s hereditary optic neuropathy. Hum Mol Genet 32(2):231–243. 10.1093/hmg/ddac190 35947995 10.1093/hmg/ddac190 PMC9840204

[147] Zhang J, Ji Y, Lu Y, Fu R, Xu M, Liu X et al (2018) Leber’s hereditary optic neuropathy (LHON)-associated ND5 12338T > C mutation altered the assembly and function of complex I, apoptosis and mitophagy. Hum Mol Genet 27(11):1999–2011. 10.1093/hmg/ddy107 29579248 10.1093/hmg/ddy107

[148] Granatiero V, Giorgio V, Calì T, Patron M, Brini M, Bernardi P et al (2016) Reduced mitochondrial Ca(2+) transients stimulate autophagy in human fibroblasts carrying the 13514A > G mutation of the ND5 subunit of NADH dehydrogenase. Cell Death Differ 23(2):231–241. 10.1038/cdd.2015.84 26206091 10.1038/cdd.2015.84 PMC4716301

[149] Galera T, Zurita F, González-Páramos C, Moreno-Izquierdo A, Fraga MF, Fernández AF et al (2016) Generation of a human iPSC line from a patient with Leigh syndrome. Stem Cell Res 16(1):63–66. 10.1016/j.scr.2015.12.005 27345786 10.1016/j.scr.2015.12.005

[150] Ma H, Folmes CDL, Wu J, Morey R, Mora-Castilla S, Ocampo A et al (2015) Metabolic rescue in pluripotent cells from patients with mtDNA disease. Nature 524(7564):234–238. 10.1038/nature14546 26176921 10.1038/nature14546

[151] Galera-Monge T, Zurita-Díaz F, Canals I, Grønning Hansen M, Rufián-Vázquez L, Ehinger JK et al (2020) Mitochondrial Dysfunction and Calcium Dysregulation in Leigh Syndrome Induced Pluripotent Stem Cell Derived Neurons. Int J Mol Sci 21(9):3191 32366037 10.3390/ijms21093191 PMC7247580

[152] Galera-Monge T, Zurita-Díaz F, Garesse R, Gallardo ME (2019) The mutation m.13513G > A impairs cardiac function, favoring a neuroectoderm commitment, in a mutant-load dependent way. J Cell Physiol 234(11):19511–19522. 10.1002/jcp.28549 30950033 10.1002/jcp.28549

[153] Lin CS, Sharpley MS, Fan W, Waymire KG, Sadun AA, Carelli V et al (2012) Mouse mtDNA mutant model of Leber hereditary optic neuropathy. Proc Natl Acad Sci 109(49):20065-70. 10.1073/pnas.1217113109 10.1073/pnas.1217113109 PMC3523873 23129651

[154] Liang M, Ji Y, Zhang L, Wang X, Hu C, Zhang J et al (2022) Leber’s hereditary optic neuropathy-associated ND6 14484T > C mutation caused pleiotropic effects on the complex I, RNA homeostasis, apoptosis and mitophagy. Hum Mol Genet 31(19):3299–3312. 10.1093/hmg/ddac109 35567411 10.1093/hmg/ddac109

[155] Wang J, Ji Y, Ai C, Chen JR, Gan D, Zhang J et al (2023) Optimized allotopic expression of mitochondrial ND6 transgene restored complex I and apoptosis deficiencies caused by LHON-linked ND6 14484T > C mutation. J Biomed Sci 30(1):63. 10.1186/s12929-023-00951-1 37537557 10.1186/s12929-023-00951-1 PMC10399063

[156] Ventura N, Rea SL, Testi R (2006) Long-lived C. elegans Mitochondrial mutants as a model for human mitochondrial-associated diseases. Exp Gerontol 41(10):974–. https://doi.org/https://doi.10.1016/j.exger.2006.06.060. 91 16945497 10.1016/j.exger.2006.06.060

[157] Shoffner JM, Brown MD, Stugard C, Jun AS, Pollock S, Haas RH et al (1995) Leber’s hereditary optic neuropathy plus dystonia is caused by a mitochondrial DNA point mutation. Ann Neurol 38(2):163–169. 10.1002/ana.410380207 7654063 10.1002/ana.410380207

[158] Rossignol R, Faustin B, Rocher C, Malgat M, Mazat J-P, Letellier T (2003) Mitochondrial threshold effects. Biochem J 370:751–762. 10.1042/BJ20021594 12467494 10.1042/BJ20021594 PMC1223225

[159] Goto Y, Horai S, Matsuoka T, Koga Y, Nihei K, Kobayashi M et al (1992) Mitochondrial myopathy, encephalopathy, lactic acidosis, and stroke-like episodes (MELAS). Neurology 42(3):545. 10.1212/WNL.42.3.545 1549215 10.1212/wnl.42.3.545

[160] Celotto AM, Chiu WK, Van Voorhies W, Palladino MJ (2011) Modes of metabolic compensation during mitochondrial disease using the Drosophila model of ATP6 dysfunction. PLoS ONE 6(10):e25823. 10.1371/journal.pone.0025823 21991365 10.1371/journal.pone.0025823 PMC3185040

[161] Tang BL (2020) Glucose, glycolysis, and neurodegenerative diseases. J Cell Physiol 235(11):7653–7662. 10.1002/jcp.29682 32239718 10.1002/jcp.29682

[162] Roca-Portoles A, Tait SWG (2021) Mitochondrial quality control: from molecule to organelle. Cell Mol Life Sci 78(8):3853–3866. 10.1007/s00018-021-03775-0 33782711 10.1007/s00018-021-03775-0 PMC8106605

[163] Walker BR, Moraes CT (2022) Nuclear-Mitochondrial Interactions. Biomolecules 12(3):427 35327619 10.3390/biom12030427 PMC8946195

[164] Bénit P, El-Khoury R, Schiff M, Sainsard-Chanet A, Rustin P (2010) Genetic background influences mitochondrial function: modeling mitochondrial disease for therapeutic development. Trends Mol Med 16(5):210–217. 10.1016/j.molmed.2010.03.001 20382561 10.1016/j.molmed.2010.03.001

[165] Maglioni S, Ventura N (2016) C. elegans as a model organism for human mitochondrial associated disorders. Mitochondrion 30. 10.1016/j.mito.2016.02.003. :117 – 25 10.1016/j.mito.2016.02.003 26906059

[166] Fox BC, Slade L, Torregrossa R, Pacitti D, Szabo C, Etheridge T et al (2021) The mitochondria-targeted hydrogen sulfide donor AP39 improves health and mitochondrial function in a C. elegans primary mitochondrial disease model. J Inherit Metab Dis 44(2):367–375. 10.1002/jimd.12345 33325042 10.1002/jimd.12345

[167] Brischigliaro M, Fernandez-Vizarra E, Viscomi C (2023) Mitochondrial Neurodegeneration: Lessons from Drosophila melanogaster Models. Biomolecules. 10.3390/biom13020378 36830747 10.3390/biom13020378 PMC9953451

[168] Christen M, Gregor A, Gutierrez-Quintana R, Bongers J, Rupp A, Penderis J et al (2024) NDUFS7 variant in dogs with Leigh syndrome and its functional validation in a Drosophila melanogaster model. Sci Rep 14(1):2975. 10.1038/s41598-024-53314-7 38316835 10.1038/s41598-024-53314-7 PMC10844639

[169] Wang A, Mouser J, Pitt J, Promislow D, Kaeberlein M (2016) Rapamycin enhances survival in a Drosophila model of mitochondrial disease. Oncotarget 7(49):80131–80139. 10.18632/oncotarget.12560 27741510 10.18632/oncotarget.12560 PMC5348310

[170] de Abreu MS, Demin KA, Kotova MM, Mirzaei F, Shariff S, Kantawala B et al (2023) Developing Novel Experimental Models of m-TORopathic Epilepsy and Related Neuropathologies: Translational Insights from Zebrafish. Int J Mol Sci 24(2). 10.3390/ijms24021530 10.3390/ijms24021530 PMC9866103 36675042

[171] Yushko LV, Kotova MM, Vyunova TV, Kalueff AV (2023) Experimental Zebrafish Models of Mitochondrial Dysfunction in the Pathogenesis of CNS Diseases. J Evol Biochem Physiol 59(6):2114–2128. 10.1134/S0022093023060170

[172] Panula P, Chen YC, Priyadarshini M, Kudo H, Semenova S, Sundvik M et al (2010) The comparative neuroanatomy and neurochemistry of zebrafish CNS systems of relevance to human neuropsychiatric diseases. Neurobiol Dis 40(1):46–57. 10.1016/j.nbd.2010.05.010 20472064 10.1016/j.nbd.2010.05.010

[173] Fichi G, Naef V, Barca A, Longo G, Fronte B, Verri T et al (2019) Fishing in the Cell Powerhouse: Zebrafish as A Tool for Exploration of Mitochondrial Defects Affecting the Nervous System. Int J Mol Sci 20(10):2409 31096646 10.3390/ijms20102409 PMC6567007

[174] Zurita Rendón O, Silva Neiva L, Sasarman F, Shoubridge EA (2014) The arginine methyltransferase NDUFAF7 is essential for complex I assembly and early vertebrate embryogenesis. Hum Mol Genet 23(19):5159–5170. 10.1093/hmg/ddu239 24838397 10.1093/hmg/ddu239 PMC4159157

[175] van de Wal MAE, Doornbos C, Bibbe JM, Homberg JR, van Karnebeek C, Huynen MA et al (2025) Ndufs4 knockout mice with isolated complex I deficiency engage a futile adaptive brain response. Biochim Biophys Acta Proteins Proteom 1873(1):141055. 10.1016/j.bbapap.2024.141055 39395749 10.1016/j.bbapap.2024.141055

[176] Leong DW, Komen JC, Hewitt CA, Arnaud E, McKenzie M, Phipson B et al (2012) Proteomic and metabolomic analyses of mitochondrial complex I-deficient mouse model generated by spontaneous B2 short interspersed nuclear element (SINE) insertion into NADH dehydrogenase (ubiquinone) Fe-S protein 4 (Ndufs4) gene. J Biol Chem 287(24):20652–20663. 10.1074/jbc.M111.327601 22535952 10.1074/jbc.M111.327601 PMC3370248

[177] Adjobo-Hermans MJW, de Haas R, Willems PHGM, Wojtala A, van Emst-de Vries SE, Wagenaars JA et al (2020) NDUFS4 deletion triggers loss of NDUFA12 in Ndufs4–/– mice and Leigh syndrome patients: A stabilizing role for NDUFAF2. Biochimica et Biophysica Acta (BBA). - Bioenergetics 1861(8):148213. 10.1016/j.bbabio.2020.148213 32335026 10.1016/j.bbabio.2020.148213

[178] Ingraham CA, Burwell LS, Skalska J, Brookes PS, Howell RL, Sheu SS et al (2009) NDUFS4: creation of a mouse model mimicking a Complex I disorder. Mitochondrion 9(3):204–210. 10.1016/j.mito.2009.02.001 19460290 10.1016/j.mito.2009.02.001 PMC2783808

[179] Reynaud-Dulaurier R, Clément R, Yjjou S, Cresson C, Saoudi Y, Faideau M et al (2024) The Blood-Brain Barrier Is Unaffected in the Ndufs4(-/-) Mouse Model of Leigh Syndrome. Int J Mol Sci 25(9). 10.3390/ijms25094828 10.3390/ijms25094828 PMC11084937 38732047

[180] Bird MJ, Wijeyeratne XW, Komen JC, Laskowski A, Ryan MT, Thorburn DR et al (2014) Neuronal and astrocyte dysfunction diverges from embryonic fibroblasts in the Ndufs4fky/fky mouse. Biosci Rep 34(6):e00151. 10.1042/bsr20140151 25312000 10.1042/BSR20140151 PMC4240023

[181] Karamanlidis G, Lee CF, Garcia-Menendez L, Kolwicz SC Jr., Suthammarak W, Gong G et al (2013) Mitochondrial complex I deficiency increases protein acetylation and accelerates heart failure. Cell Metab 18(2):239–250. 10.1016/j.cmet.2013.07.002 23931755 10.1016/j.cmet.2013.07.002 PMC3779647

[182] Chen B, Hui J, Montgomery KS, Gella A, Bolea I, Sanz E et al (2017) Loss of Mitochondrial Ndufs4 in Striatal Medium Spiny Neurons Mediates Progressive Motor Impairment in a Mouse Model of Leigh Syndrome. Front Mol Neurosci 10:265. 10.3389/fnmol.2017.00265 28883788 10.3389/fnmol.2017.00265 PMC5573716

[183] Quintana A, Kruse SE, Kapur RP, Sanz E, Palmiter RD (2010) Complex I deficiency due to loss of Ndufs4 in the brain results in progressive encephalopathy resembling Leigh syndrome. Proc Natl Acad Sci U S A 107(24):10996–11001. 10.1073/pnas.1006214107 20534480 10.1073/pnas.1006214107 PMC2890717

[184] Schirris TJJ, Rossell S, de Haas R, Frambach S, Hoogstraten CA, Renkema GH et al (2021) Stimulation of cholesterol biosynthesis in mitochondrial complex I-deficiency lowers reductive stress and improves motor function and survival in mice. Biochim Biophys Acta Mol Basis Dis 1867(4):166062. 10.1016/j.bbadis.2020.166062 33385517 10.1016/j.bbadis.2020.166062

[185] Jain IH, Zazzeron L, Goldberger O, Marutani E, Wojtkiewicz GR, Ast T et al (2019) Leigh Syndrome Mouse Model Can Be Rescued by Interventions that Normalize Brain Hyperoxia, but Not HIF Activation. Cell Metab 30(4):824–32e3. 10.1016/j.cmet.2019.07.006 31402314 10.1016/j.cmet.2019.07.006 PMC6903907

[186] Warwick AM, Bomze HM, Wang L, Hao Y, Stinnett SS, Gospe SM 3 (2024) Hypoxia-mediated rescue of retinal ganglion cells deficient in mitochondrial complex I is independent of the hypoxia-inducible factor pathway. Sci Rep 14(1):24114. 10.1038/s41598-024-75916-x 39406814 10.1038/s41598-024-75916-x PMC11480089

[187] Forbes JM, Ke BX, Nguyen TV, Henstridge DC, Penfold SA, Laskowski A et al (2013) Deficiency in mitochondrial complex I activity due to Ndufs6 gene trap insertion induces renal disease. Antioxid Redox Signal 19(4):331–343. 10.1089/ars.2012.4719 23320803 10.1089/ars.2012.4719

[188] Walker BR, Theard LM, Pinto M, Rodriguez-Silva M, Bacman SR, Moraes CT (2024) Restoration of defective oxidative phosphorylation to a subset of neurons prevents mitochondrial encephalopathy. EMBO Mol Med 16(9):2210–2232. 10.1038/s44321-024-00111-4 39169163 10.1038/s44321-024-00111-4 PMC11392956

[189] Kim C, Potluri P, Khalil A, Gaut D, McManus M, Compton S et al (2017) An X-chromosome linked mouse model (Ndufa1(S55A)) for systemic partial Complex I deficiency for studying predisposition to neurodegeneration and other diseases. Neurochem Int 109:78–93. 10.1016/j.neuint.2017.05.003 28506826 10.1016/j.neuint.2017.05.003

[190] Felici R, Lapucci A, Cavone L, Pratesi S, Berlinguer-Palmini R, Chiarugi A (2015) Pharmacological NAD-Boosting Strategies Improve Mitochondrial Homeostasis in Human Complex I-Mutant Fibroblasts. Mol Pharmacol 87(6):965–971. 10.1124/mol.114.097204 25788480 10.1124/mol.114.097204

[191] Iannetti EF, Smeitink JAM, Willems P, Beyrath J, Koopman WJH (2018) Rescue from galactose-induced death of Leigh Syndrome patient cells by pyruvate and NAD(). Cell Death Dis 9(11):1135. 10.1038/s41419-018-1179-4 30429455 10.1038/s41419-018-1179-4 PMC6235972

[192] Borna NN, Kishita Y, Shimura M, Murayama K, Ohtake A, Okazaki Y (2024) Identification of a novel MT-ND3 variant and restoring mitochondrial function by allotopic expression of MT-ND3 gene. Mitochondrion 76:101858. 10.1016/j.mito.2024.101858 38437941 10.1016/j.mito.2024.101858

[193] Bonnet C, Augustin S, Ellouze S, Bénit P, Bouaita A, Rustin P et al (2008) The optimized allotopic expression of ND1 or ND4 genes restores respiratory chain complex I activity in fibroblasts harboring mutations in these genes. Biochimica et Biophysica Acta (BBA) -. Mol Cell Res 1783(10):1707–1717. 10.1016/j.bbamcr.2008.04.018 10.1016/j.bbamcr.2008.04.018 18513491

[194] Melcher M, Danhauser K, Seibt A, Degistirici Ö, Baertling F, Kondadi AK et al (2017) Modulation of oxidative phosphorylation and redox homeostasis in mitochondrial NDUFS4 deficiency via mesenchymal stem cells. Stem Cell Res Ther 8(1):150. 10.1186/s13287-017-0601-7 28646906 10.1186/s13287-017-0601-7 PMC5482938

[195] Navaratnarajah T, Bellmann M, Seibt A, Anand R, Degistirici Ö, Meisel R et al (2022) Mesenchymal stem cells improve redox homeostasis and mitochondrial respiration in fibroblast cell lines with pathogenic MT-ND3 and MT-ND6 variants. Stem Cell Res Ther 13(1):256. 10.1186/s13287-022-02932-x 35715829 10.1186/s13287-022-02932-x PMC9205113

[196] Heiduschka S, Prigione A (2025) iPSC models of mitochondrial diseases. Neurobiol Dis 207:106822. 10.1016/j.nbd.2025.106822 39892770 10.1016/j.nbd.2025.106822

[197] Wilkins HM, Carl SM, Swerdlow RH (2014) Cytoplasmic hybrid (cybrid) cell lines as a practical model for mitochondriopathies. Redox Biol 2:619–631. https://doi.org/https://doi.org/10.1016/j.redox.2014.03.006 25460729 10.1016/j.redox.2014.03.006 PMC4297942

[198] Giacomotto J, Ségalat L (2010) High-throughput screening and small animal models, where are we? Br J Pharmacol 160(2):204–. 10.1111/j.1476-5381.2010.00725.x. 16 20423335 10.1111/j.1476-5381.2010.00725.x PMC2874843

[199] Ősz F, Nazir A, Takács-Vellai K, Farkas Z (2025) Mutations of the Electron Transport Chain Affect Lifespan and ROS Levels in C. elegans. Antioxidants 14(1):76 39857410 10.3390/antiox14010076 PMC11761250

[200] Adjobo-Hermans MJW, de Haas R, Willems P, Wojtala A, van Emst-de Vries SE, Wagenaars JA et al (2020) NDUFS4 deletion triggers loss of NDUFA12 in Ndufs4(-/-) mice and Leigh syndrome patients: A stabilizing role for NDUFAF2. Biochim Biophys Acta Bioenerg 1861(8):148213. 10.1016/j.bbabio.2020.148213 32335026 10.1016/j.bbabio.2020.148213

[201] Tolle I, Tiranti V, Prigione A (2023) Modeling mitochondrial DNA diseases: from base editing to pluripotent stem-cell‐derived organoids. EMBO Rep 24(4):EMBR202255678. 10.15252/embr.202255678 10.15252/embr.202255678 PMC10074100 36876467

[202] Lyu L, Qie B, He Y, Chen F, Liu B (2025) Advances in gene therapy for mitochondrial genetic disorders: current status and clinical implementation challenges. J Transl Med 23(1):1415. 10.1186/s12967-025-07420-3 41437048 10.1186/s12967-025-07420-3 PMC12729811

[203] Wen H, Deng H, Li B, Chen J, Zhu J, Zhang X et al (2025) Mitochondrial diseases: from molecular mechanisms to therapeutic advances. Signal Transduct Target Ther 10(1):9. 10.1038/s41392-024-02044-3 39788934 10.1038/s41392-024-02044-3 PMC11724432

[204] Fearnley IM, Walker JE (1992) Conservation of sequences of subunits of mitochondrial complex I and their relationships with other proteins. Biochimica et Biophysica Acta (BBA). - Bioenergetics 1140(2):105–134. 10.1016/0005-2728(92)90001-I 10.1016/0005-2728(92)90001-i 1445936

