# Supplementary material for: Mitochondrial complex I deficiency-associated diseases and models
Source: Cell Mol Life Sci. 2026 Jun 10;83(1):249. doi: 10.1007/s00018-026-06169-2 (PMC13249995; doi:10.1007/s00018-026-06169-2)
Supplement: Supplementary file 1 — Supplementary Material 1 [file 18_2026_6169_MOESM1_ESM.docx]

Cellular and Molecular Life Sciences

Mitochondrial Complex I deficiency-associated diseases and models

Lena Jentsch^1,2^, Natascia Ventura^1,2,3,4^ *

**Affiliations**

1. Institute of Clinical Chemistry and Laboratory Diagnostic, Medical Faculty, Heinrich Heine University Düsseldorf, Moorenstraße 5, 40225 Düsseldorf
2. IUF – Leibniz Research Institute for Environmental Medicine, Auf’m Hennekamp 50, 40225 Düsseldorf
3. Institute of Cell Biology, Heinrich Heine University Düsseldorf, Universitätsstraße 1, 40225 Düsseldorf

* Corresponding author E-mail address: [natascia.ventura@hhu.de](mailto:natascia.ventura@hhu.de)

**Supplementary Table 1.** Table of mitochondrial CI genes with details on main reported pathogenic variants and respective key clinical features, ordered according to their location in CI modules

| **Gene** | **Reported pathogenic variants** | **Key clinical features** |
| --- | --- | --- |
| **N-module** | | |
| ***NDUFV1*** | 175C>T – 1268C>T [53]  1022C>T [36, 53]  640G>A – IVS8+41A>C [47]  1294C>C – 989-990del [47]  611A>G – 616T>G [47]  770G>A – 632T>C [79]  1129G>A[51]  365C>T – 565T>C [56]  1157G>A – 1080G>A[52]  640A>G – 248T>C [80] | Early onset, fatal LS/LLS [53]  Leukoencephalomyopathy [36, 53, 55]  Lactic acidosis, hypotonia, ataxia [47]  Mild leukoencephalopathy [79]  Late onset leukoencephalopathy [56]  Hypotonia, loss of motor function[52]  Paralysis, loss of balance [80] |
| ***NDUFV2*** | IVS2+5_+8delGTAA [57]  580G>A[58]  580G>A – 427C>T[58] | Cardiomyopathy, encephalopathy[57, 59]  Lactic acidosis, loss of motor function, developemental delay [58] |
| ***NDUFS1*** | 664-666del – 755A>G [47]  721C>T [47]  2119A>G [47]  1564C>A [36, 63]  691C>G [64]  1855G>A - 1669C>T[61]  1222C>T[61] [67]  631-633del – 683T>C[61]  1783A>G [62]  61C>T – 584T>C [65] | Lactic acidosis, hypotonia [47]  LS [47, 64]  Leukoencephalopathy [36, 63]  LS [64]  Hypotonia [67]  LLS [61]  Leukoencephalopathy [62]  LS [65] |
| ***NDUFS4*** | 44G>A [63]  289-290del[69]  316C>T [69]  IVS1nt-1G>A [60]  462delA [68]  350+5G>A[81] | LS, cardiomyopathy [63]  LLS [69]  LS [60]  LS [68]  Encephalopathy [81] |
| ***NDUFS6*** | 186+2T>A [70]  344G>A[73]  344G>T[71]  309+5G>A – 343T>C [72] | Lactic acidosis, respiratory failure [70]  Lactic acidosis[73]  Lactic acidosis, brain abnormalities [71]  LS [72] |
| ***NDUFA2*** | 208+5G>A [74]  134A>C [77]  134A>C – 225del[77] | LS, cardiomyopathy [74]  Leukoencephalopathy[77] |
| ***NDUFA8*** | 325G>A [36]  239C>T[78] | Neonatal hypotonia, epilepsy [36]  Developmental delay, microcephaly, epilepsy [78] |
| ***NDUFA12*** | 178C>T [75, 76]  121dupG [75]  4G>T [75]  253G>T [75]  83del [75]  86G>A [75]  395delA[75]  224G>A [75] | LS [76],  Scoliosis, lactic acidosis, isolated optic atrophy, LS, LLS [75] |
| **Q-module** | | |
| ***NDUFS2*** | 671C>T [36] 683G>A [82]  686C>A [82]  1237T>C [82]  252T>G – 311A>G [83]  1237T>C – 552delC [84] | Neonatal hypotonia, epilepsy [36]  Cardiomyopathy, hypotonia, lactic acidosis [82]  LS, optic atrophy [83]  Lactic acidosis, liver pathology [84] |
| ***NDUFS3*** | 434C>T – 595C>T [85]  418C>T – 595C>T [87] 419G>A – 381+6T>C [86] | Late onset LS [85]  LS [87] mild LS [86] |
| ***NDUFS7*** | -3+1AAGAdel,TCTins [36]  364G>A[88] | Neonatal cardiomyopathy [36]  LS [88] |
| ***NDUFS8*** | 460G>A [51]  236C>T [67]  236TC>T – 305G>A [89]  304C>T [90] | Encephalopathy [51]  Lactic acidosis, respiratory failure [67]  LS [89]  Stroke-like episodes, motor regression[90] |
| ***NDUFA6*** | 191G>C – 265G>T[91]  331-332del 3G>A 309del – 355del | Lactic acidosis, hypotonia, motor regression, optic atrophy [91] |
| **P-module** | | |
| ***ND1*** | 3460GC>AT [95]  3796A>G [93]  3697G>A[94]  3946G>A[94]  3949T>C[94]  3460G>A[96]  3866T>C [114]  3761C>A[109]  3911A>G [116]  3955G>A [99]  3571-372insC [97] | LHON [95]  Dystonia [93]   MELAS [94]  LS [96] LHON [114]  Late onset hearing loss, neuronal regression [109]  Neurodevelopmental delay, hearing loss, hypotonia[116]  LS[99]  MELAS [97] |
| ***ND2*** | 4917A>G [98]  5182C>T [112]  5178C>A [113] | LHON [98]  Cardiomyopathy[112]  Diabetes mellitus [113] |
| ***ND3*** | 10191T>C[36] 10158T>C[36, 70]  10158T>C[100]  10191T>C[100]  10197G>A [101] | LS [36]  Infantile encephalopathy, LS, LLS [100]  MELAS/LS overlap[101] |
| ***ND4*** | 11777C>A[36]  11778G>A [114]  12058A>C[116]  11777G>A [96]  12083G [148]  11406T>A [102] | LS [36]  LHON [114]  Neurodevelopmental delay, hearing loss, hypotonia[116]  LHON [96] LHON [148]  MELAS[102] |
| ***ND4L*** | 10663C [148]  10680G>A [117] | LHON [117, 148] |
| ***ND5*** | 13513G>A[36, 103]  13514A>G[36]  13708G>A [98]  12338T>C [150] | LS [36]  LHON, nephropathy [103]  LHON[98] LHON [150] |
| ***ND6*** | 14487T>C[36, 107]  14453G>A[106]  14484T>C [96]  14512-14513del [109]  14502T>C[108] | LS [36, 107]  MELAS[106]  LHON[96]  Late onset myopathy [109] Mild LHON [108] |
| ***NDUFA13*** | 170G>A[111]  164G>C[111]  187G>A – 170G>A[111]  107T>C – 94+1G>A[111]  107T>C[111] | Neurodevelopmental delay, spasticity, hypotonia, epilepsy [111] |
| ***NDUFC2*** | 346-*7del [110] 173A>T[110] | LS[110] |
